# Small, correlated changes in synaptic connectivity may facilitate rapid motor learning

**DOI:** 10.1038/s41467-022-32646-w

**Published:** 2022-09-02

**Authors:** Barbara Feulner, Matthew G. Perich, Raeed H. Chowdhury, Lee E. Miller, Juan A. Gallego, Claudia Clopath

**Affiliations:** 1 Department of Bioengineering, Imperial College London, London, UK; 2 Département de neurosciences, Université de Montréal, Montréal, QC Canada; 3 Department of Bioengineering, University of Pittsburgh, Pittsburgh, PA USA; 4 Department of Neuroscience, Northwestern University, Evanston, IL USA; 5 Department of Biomedical Engineering, Northwestern University, Evanston, IL USA; 6 Department of Physical Medicine and Rehabilitation, Northwestern University, and Shirley Ryan Ability Lab, Chicago, IL USA

**Keywords:** Neuroscience, Computational neuroscience, Learning and memory, Motor control

## Abstract

Animals rapidly adapt their movements to external perturbations, a process paralleled by changes in neural activity in the motor cortex. Experimental studies suggest that these changes originate from altered inputs (H_input_) rather than from changes in local connectivity (H_local_), as neural covariance is largely preserved during adaptation. Since measuring synaptic changes in vivo remains very challenging, we used a modular recurrent neural network to qualitatively test this interpretation. As expected, H_input_ resulted in small activity changes and largely preserved covariance. Surprisingly given the presumed dependence of stable covariance on preserved circuit connectivity, H_local_ led to only slightly larger changes in activity and covariance, still within the range of experimental recordings. This similarity is due to H_local_ only requiring small, correlated connectivity changes for successful adaptation. Simulations of tasks that impose increasingly larger behavioural changes revealed a growing difference between H_input_ and H_local_, which could be exploited when designing future experiments.

## Abstract (web summary)

How animals are able to rapidly adapt their behaviour to changing environmental demands remains poorly understood. Here, the authors use a modelling approach to show that synaptic plasticity in motor cortex may underlie rapid motor learning, demonstrating that small, correlated connectivity changes that preserve neural covariance are highly effective in driving behavioural adaptation.

## Introduction

Animals, particularly primates, can perform a great variety of behaviours, which they are able to adapt rapidly in the face of changing conditions. Since behavioural adaptation can happen even after a single failed attempt^1^, the neural populations driving this process must be able to adapt equally fast. How this occurs remains unexplained^2^. Rapid motor learning is typically studied using external perturbations such as a visuomotor rotation (VR), which rotates the coordinates of the visual feedback with respect to those of the movement. Both humans and monkeys can learn to compensate for the resulting error between actual and expected visual feedback in a few tens of trials^3,4^. This behavioural adaptation is accompanied by changes in the activity of neurons in primary motor cortex (M1)^5^, and the upstream dorsal premotor cortex (PMd)^3^. It is unclear whether these neural activity changes are mediated by synaptic weight changes within the motor cortices or are driven by altered inputs from even further upstream areas.

When learning a skill over many days, behavioural improvements are paralleled by rewiring between M1 neurons^6–9^. This seems not to be the case for rapid learning: throughout VR adaptation, the statistical interactions across neural populations in both M1 and PMd remain largely preserved^10^. These preserved interactions rule out any large synaptic changes within the motor cortices, as they would cause these models to degrade^11,12^. Instead, rapid VR adaptation may be driven by the cerebellum^13–16^ and/or posterior parietal cortex^17,18^.

A pioneering Brain Computer Interface (BCI) study cast further doubt that significant synaptic changes occurring within M1 are necessary for rapid learning^19,20^. In that study, monkeys controlled a computer cursor linked by a “decoder” to the activity of recorded M1 neurons. After learning to use a decoder that used the natural “intrinsic” mapping of neural activity onto cursor movements, the monkeys were exposed to one of two types of perturbations. When faced with a new decoder that preserved the statistical interactions (i.e., neural covariance) across M1 neurons, the monkeys could master it within minutes. In stark contrast, if the new decoder required changes in the neural covariance (an “out of manifold” perturbation), they could not learn it within one session—in fact, it required a progressive training procedure spanning just over nine days on average^21^.

Recording large scale synaptic changes in vivo remains challenging and has not been achieved during rapid motor learning. Alternatively, recurrent neural network (RNN) models offer an exciting yet unexplored opportunity to test the effect of synaptic changes (in the model) on simulated activity during motor learning. RNNs trained on motor, cognitive and BCI tasks exhibit many striking similarities with the activity of neural populations recorded in animal studies^22–28^, suggesting a fundamental similarity between the two. Previous work using RNNs to model the BCI experiment described above^19^ showed that network covariance can be highly preserved even when learning is happening through weight changes within the network^29^. Thus, contrary to intuition, functionally relevant synaptic weight changes may not necessarily lead to measurable changes in statistical interactions across neurons^30^. As a consequence, synaptic changes within PMd and M1 during VR adaptation may be very hard to identify through the analysis of neural population recordings.

**Fig. 1:**
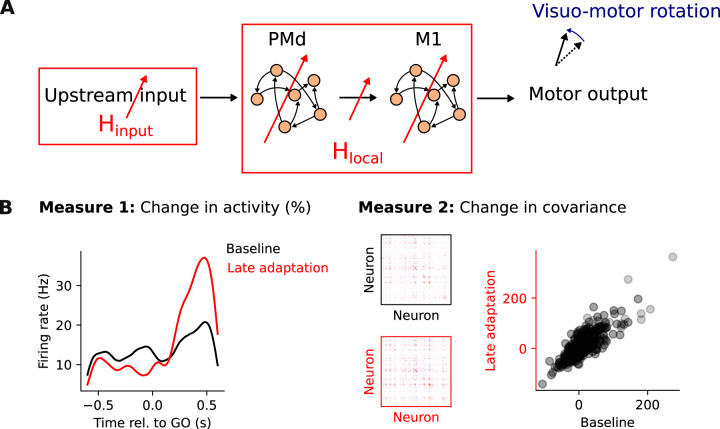
Competing hypotheses to explain where learning happens during a visuomotor rotation task. **A** To study the processes mediating motor cortical activity changes during adaptation in a visuomotor rotation task, we analyze and model the activity of neural populations within dorsal premotor cortex (PMd) and primary motor cortex (M1). We compare two hypotheses: plasticity upstream of PMd/M1 (H_input_) and plasticity within PMd/M1 (H_local_). **B** Measures to quantify the changes in neural activity following adaptation: (1) relative change in trial-averaged single neuron activity; (2) change in neural covariance. Both measures compare baseline trials to late adaptation trials, after monkeys had adapted to the task. Data from a representative session from Monkey C.

Here, we used RNN models to test whether VR adaptation might be mediated by synaptic changes within PMd and M1, yet with largely preserved neural covariance within these areas. We addressed this question by asking how adaptation based on connection weight changes within PMd and M1 (H_local_) alters network activity compared to the corresponding activity changes if VR adaptation is based on altered inputs from upstream areas (H_input_)^10,13–18^ (Fig. 1A). To validate our modelling results, we compared our simulations to experimental recordings from PMd and M1 populations during the same VR task^10^.

Under H_local_, the changes in covariance following VR adaptation only slightly exceeded those under H_input_ and were comparable to experimental observations. Thus, when using neural population recordings alone, it may be more challenging to disentangle these two hypotheses than previously thought. Moreover, for both H_input_ and H_local_, the learned connectivity changes were small and highly coordinated, which made them surprisingly robust to noise. To identify additional differences between H_input_ and H_local_, we examined learning during paradigms requiring larger behavioural changes. Covariance changes were larger for these paradigms in both PMd and M1 under H_input_, but only in M1 under H_local_, thus providing a possible way to distinguish between the two hypotheses in future experiments. Our findings have implications for the interpretation of neural activity changes observed during learning, and suggest that tasks eliciting larger behavioural changes may be necessary to elucidate how neural populations adapt their activity during rapid learning.

## Results

To understand whether motor adaptation could be driven by synaptic changes within PMd and M1, we simulated a VR adaptation task using a modular RNN that modelled these two areas, and compared the resulting changes in network activity to those of neural population recordings from PMd and M1 during the same VR task^10^. We quantified neural activity changes both in the experimental data and in the model using two measures (Fig. 1B): (1) the relative change in trial-averaged single neuron activity, and (2) the change in neural covariance (“Methods”). Combined, they capture aspects of single neuron as well as population-wide activity changes during adaptation.

### Small but measurable changes in neural activity within PMd and M1 during VR adaptation

**Fig. 2:**
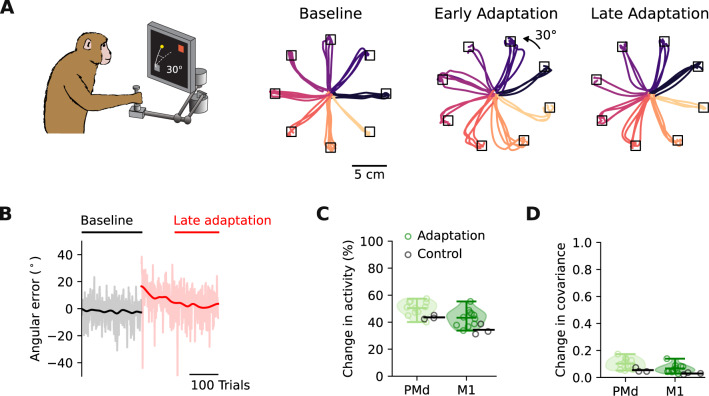
Small but measurable changes in neural activity within PMd and M1 during VR adaptation. **A** Hand trajectories during the first 30 trials of the baseline, and the early adaptation epoch (first 150 adaptation trials), and the last 30 trials of the late adaptation epoch (last 150 adaptation trials). Trajectories are colour-coded by target. Data from Monkey C. **B** Angular error of the hand trajectories for the example session in **A** has the typical time course of adaptation. **C** Change in trial-averaged activity following adaptation. Data pooled across all sessions from the two monkeys for PMd and M1 separately (green markers, 11 sessions, 5 Monkey C, 6 Monkey M). Control sessions during which no perturbation was applied are shown for comparison (black markers, 3 sessions, 1 Monkey C, 2 Monkey M). Shaded area and horizontal bars, data distribution with mean and extrema (*n* = 11 experimental sessions). **D** Change in covariance following adaptation. Same format as (**C**). The monkey image was created by Carolina Massumoto who gave permission to use it under CC-BY license.

Monkeys were trained to perform an instructed delay task, in which they reached to one of eight visual targets using a planar manipulandum to receive a reward (“Methods”). After performing a block of unperturbed reaches (200–243 trials, depending on the session), visual feedback about the position of the hand was rotated by 30°, either clockwise or counterclockwise, depending on the session. Monkeys adapted rapidly to these perturbations: the curved reaches observed immediately after the perturbation onset became straighter after tens of trials, with the hand trajectories in the second (late) half of the adaptation block becoming more similar to the baseline trajectories (Fig. 2A). The angular error quantifying the difference between initial reach direction and target location decreased during adaptation (Fig. 2B). This error curve followed a similar trend for clockwise and counterclockwise perturbations, allowing us to analyze the different perturbations together.

Behavioural adaptation was accompanied by changes in neural activity within both PMd and M1 (Fig. 2C)^10^. These changes exceeded those during control sessions, where no perturbation was applied (Fig. 2C black; linear mixed model analysis: *t* = 4.4, *P* = 0.0017). The amount of change was greater within PMd than M1 (*t* = 8.9, *P* < 0.0001). We also found small but detectable changes in neural covariance during VR perturbation, suggesting that the statistical interactions among neurons change slightly during adaptation (Fig. 2D). Again, these changes exceeded those of the control sessions (Fig. 2D black; *t* = 2.6, *P* = 0.026).

### A modular recurrent neural network model to study VR adaptation

**Fig. 3:**
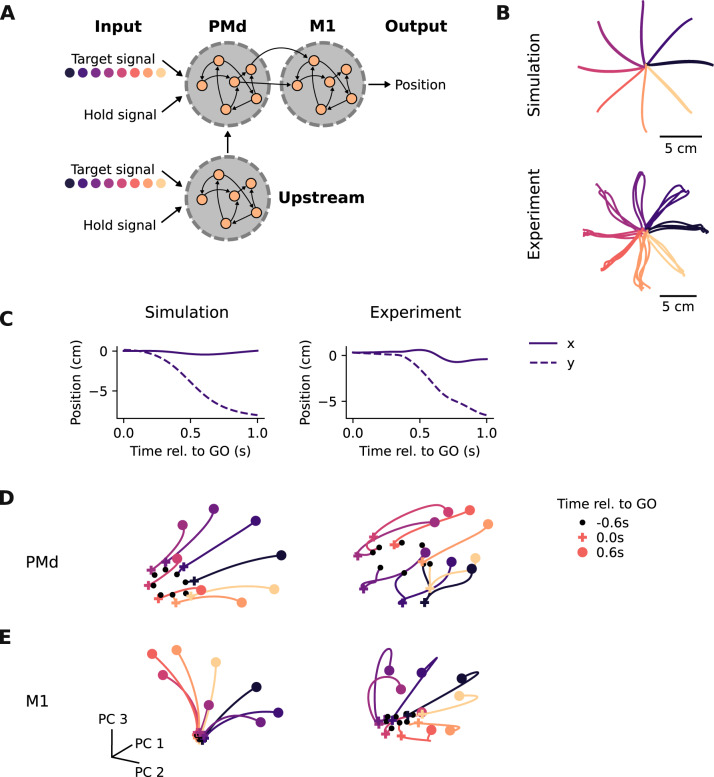
A modular recurrent neural network model to study VR adaptation. **A** A modular RNN that models key motor cortical areas to study adaptation. **B** Simulated (top) and actual (bottom) hand trajectories during 30 reaches to each target taken from one session from Monkey C. **C** Example simulated and actual hand trajectories to one target. Note the similarity in kinematics between the model and the experimental data. **D** Simulated PMd population activity recapitulates key features of actual PMd population activity. Neural trajectories extend from 600 ms before the go cue (black dots) to 600 ms after the go cue (coloured dots); go cue is indicated with coloured crosses. Reaching targets are colour-coded as in **B**. **E** Same as (**D**) for M1.

To test whether experimentally observed changes in motor cortical activity could be driven by rapid synaptic plasticity^9^ within PMd and M1, we trained a modular RNN model^23,27^ to perform the centre-out reaching task that we studied experimentally. To mimic broadly the hierarchical architecture of the motor cortical pathways, input signals were sent to the PMd module which then projected to the M1 module to produce the final output signal (Fig. 3A; “Methods”). After initial training on the task, the model produced correct reaching trajectories to each of the eight different targets (Fig. 3B and Supplementary Fig. 1). These RNN-controlled movements had the same dynamics as those of monkeys (Fig. 3C). Furthermore, *Principal Component Analysis* revealed that the population activity of the PMd and M1 network modules was similar to that of the corresponding recorded neural populations (Fig. 3D, E). We used Procrustes analysis^31^ to quantify this apparent similarity between model and experimental population activity (Supplementary Fig. 2). This analysis confirmed that the modular RNN captured the area-specific features in the neural data accurately, as the PMd and M1 modules better explained neural data from the respective brain area compared to a cross-area control (Supplementary Fig. 2).

### Motor adaptation through altered inputs matches neural recordings

**Fig. 4:**
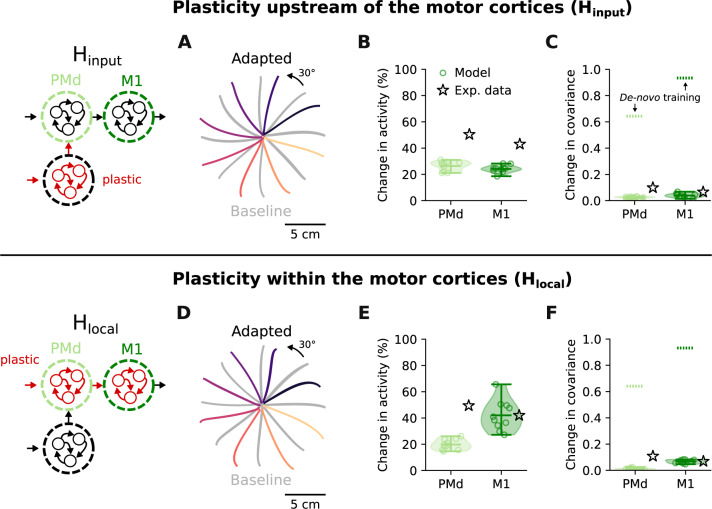
Activity changes following learning upstream (H_input_) and within (H_local_) the motor cortices. **A** Hand trajectories after learning under H_input_ (coloured traces; baseline trajectories are shown in grey). **B** Changes in trial-averaged activity following adaptation under H_input_ (green markers) for PMd and M1, and reference mean experimental values (black stars; same data as presented in Fig. 2). Shaded area and horizontal bars, data distribution with mean and extrema (*n* = 10 network initializations). **C** Change in covariance following adaptation under H_input_, and reference values for change in covariance following the initial *de-novo* training (dashed lines). Data presented as in **B**. **D** Hand trajectories after learning under H_local_. **E** Change in trial-averaged activity following adaptation under H_local_. **F** Change in covariance following adaptation under H_local_. Data in **D**–**F** are presented as in **A**–**C**. Source data.

After having verified that our modular RNN recapitulates the key aspects of PMd and M1 population activity during reaching, we simulated the VR adaptation experiment. The model was retrained to produce trajectories rotated by 30°, replicating the perturbation monkeys had to counteract. Having full control of the location of learning-related changes, we first constrained it to happen upstream of PMd (H_input_). As anticipated from previous modelling^18^ and experimental work^10^, changes in areas upstream of the motor cortices can lead to successful adaptation: the hand trajectories produced after learning were correctly rotated by 30° to counteract the perturbation (Fig. 4A).

When examining the activity of each of the PMd and M1 modules, the relative change in network activity was similar in magnitude to the changes observed in the corresponding neural population recordings (Fig. 4B and Supplementary Fig. 3). PMd activity changed slightly more than M1 activity (Fig. 4B), indicating a relation between the two modules that was also present in the experimental data (Fig. 2C). With respect to interactions between neurons, covariance within each module was strongly preserved (Fig. 4C), as was the case for the experimental data (Fig. 2D). VR adaptation through altered inputs to the motor cortices thus is very similar to the neural activity changes observed in vivo.

### Learning through plastic changes within PMd and M1 modules occurs despite preserved the covariance

Our simulation results so far are consistent with experimental^10,13–15^ and modelling^18^ studies proposing that VR adaptation is mediated by regions upstream of the motor cortices. But can our model rule out the alternative that adaptation is instead implemented by recurrent connectivity changes within PMd and M1 (H_local_)?

To address this question, we implemented H_local_ by constraining learning to happen only within PMd and M1, a process which also led to successful adaptation (Fig. 4D). Interestingly, the activity changes produced under H_local_ differed both from those of H_input_ and the experimental data: there were larger changes in the M1 module than in the PMd module (Fig. 4E). However, learning based on recurrent weight changes within PMd and M1 did not lead to large changes in covariance, which was largely preserved (Fig. 4F), virtually as much as when no local plasticity was allowed (H_input_) (Fig. 4C). Thus, the intuition that preserved covariance should be interpreted as a sign of stable underlying connectivity may be misleading.

### Small but coordinated connectivity changes enable motor adaptation

**Fig. 5:**
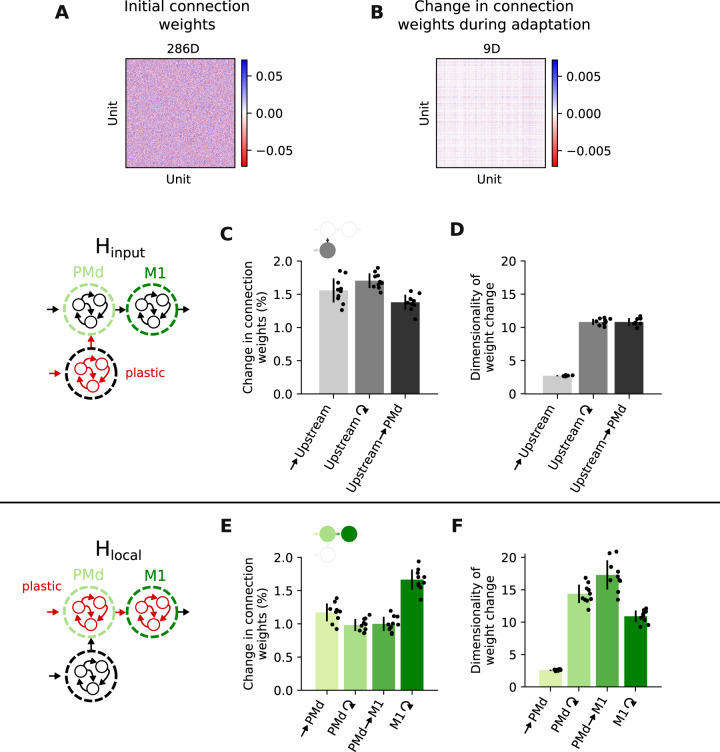
Small but coordinated connectivity changes enable motor adaptation. **A** Example connection weights for the M1 module after initial training. Top: estimated dimensionality. **B** Example changes in M1 connection weights following VR adaptation under H_local_. Same format as (**A**). **C** Change in connection weights following adaptation under H_input_. Each bar summarizes results for either one module of the network or a set of cross-module connections; bars and error bars, mean and s.d. (*n* = 10 network initializations). **D** Estimated dimensionality of connection weight changes for each network module and cross-module connections; data presented as in (**C**). **E** Change in connection weights following adaptation under H_local_. **F** Dimensionality of connection weight changes under H_local_. Data in **E**, **F** are presented as in **C**, **D**, respectively. Source data.

We wished to understand how the model can adapt to the VR perturbation by changing the recurrent connectivity within the PMd and M1 modules without altering their covariance. Interestingly, the connectivity changes under both H_local_ and H_input_ (Fig. 5A, B) were small relative to experimentally observed synaptic changes^32^: an average weight change of 1–2% was sufficient regardless of whether they happened upstream of (Fig. 5C and Supplementary Fig. 4) or within the motor cortical modules (Fig. 5E and Supplementary Fig. 5). These changes were smaller than those observed during initial training (4–31%), when the model learned to perform the reaching task from random connection weights (Supplementary Fig. 6). Thus, “functional connectivity” within the PMd and M1 modules, as measured here by their covariance, may be largely preserved after VR adaptation under H_local_ because network connection weights change very little (Supplementary Fig. 7).

We next studied how such small changes in connection weights could nevertheless drive effective behavioural adaptation. Recent studies seeking to relate RNN activity and connectivity have highlighted the importance of low-dimensional structures in connectivity, showing their explanatory power for understanding how tasks are solved^33–36^. Inspired by this work, we looked for low-dimensional structure in the connectivity changes emerging in the model during adaptation (“Methods”). Our analysis revealed that the connectivity change patterns of all plastic modules were low-dimensional, independent of where learning happened (Fig. 5B, D, F). We thus hypothesized that the small changes were effective because they were low-dimensional. To test this, we examined how random changes in the connection weights (noise), which are inherently high-dimensional, would affect the behaviour.

### Low-dimensional connectivity changes are highly robust to noise

**Fig. 6:**
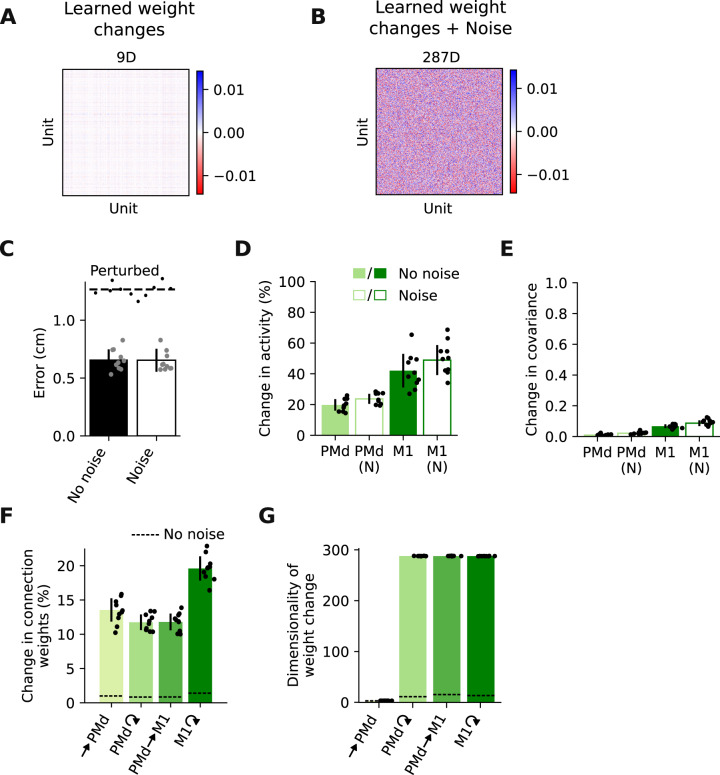
Low-dimensional connectivity changes are highly robust to noise. **A** Example changes in M1 connection weights following VR adaptation. Same data as in Fig. 5B. **B** Same connection weight changes as in **A**, but with random connectivity changes added. Note the dramatic increase in the dimensionality of the connection weight changes. **C** Root mean squared error between target and produced hand trajectories following adaptation in models with and without random weight changes; bars and error bars, mean and s.d. (*n* = 10 network initializations), as in all the panels in this figure. Dashed line, error under VR perturbation without any learning. **D** Change in trial-averaged activity for PMd and M1 module, without (solid) and with (empty) random weight changes. **E** Change in covariance. Same format as (**D**). **F** Change in connection weights for each network module and cross-module connections in models with (green bars) and without (dashed lines) noise in connectivity. **G** Dimensionality of connection weight changes. Same format as (**F**).

For both H_local_ and H_input_, the learned connectivity changes in the model were small and low-dimensional. When considering the biological plausibility of our model, this observation raises the question of how such small connectivity changes could compete with ongoing synaptic fluctuation, which is a known challenge for actual brains^37–40^. To test the hypothesis that the low-dimensionality of the learned connectivity changes is what makes them highly effective, we tested how adding synaptic fluctuations, which are inherently high-dimensional, would affect motor output. Simulating synaptic fluctuations by applying random perturbations to the learned connectivity changes increased the dimensionality of the weight changes (Fig. 6B, G; “Methods”), but did not lead to any observable change in reaching kinematics (Fig. 6C) or network activity (Fig. 6D, E). This was the case even though the applied random perturbations in connectivity were ten times bigger in magnitude than the learned connectivity changes (Fig. 6F), completely masking them (Fig. 6A, B). Therefore, our model not only suggests that VR adaptation can be implemented based on coordinated synaptic weight changes within PMd and M1, but also that this type of learning would be highly effective due to its robustness to synaptic fluctuation.

### Larger visuomotor rotations allow for a clearer distinction between H_input_ and H_local_

Although neural activity changes during VR adaptation were better reproduced by a model in which learning happens upstream of the motor cortices (H_input_), activity changes following learning through weight changes within the motor cortices (H_local_) were also in good agreement with the experimental data. To verify that the stable covariance (Fig. 4C, F) is not a general feature of the model but reflects task-specific demands, we modelled tasks for which we would expect larger changes.

**Fig. 7:**
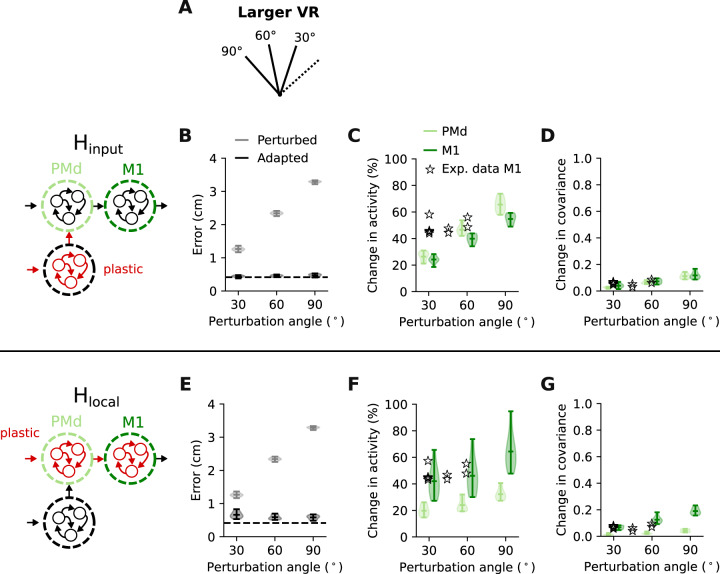
Larger visuomotor rotations allow for a better distinction between H_input_ and H_local_. **A** To verify that the modelled perturbation does not always produce small activity changes, we tested adaptation to larger VR perturbations (60° and 90°). **B** Root mean squared error between target and produced hand trajectories without (grey) and with learning (black) under H_input_. Dashed line, error after initial training, with no perturbation applied; shaded area and horizontal bars, data distribution with mean and extrema (*n* = 10 network initializations). Same data presentation in all panels. **C** Change in trial-averaged activity for PMd and M1 under H_input_. A few experimental sessions from Monkey C with larger rotations are shown as comparison (stars). **D** Change in covariance following adaptation. Data are presented as in C. A few experimental sessions from Monkey C with larger rotations are again shown as comparison (stars). Note the similarity between PMd (light green) and M1 (dark green) across all rotation angles. **E** Error without (grey) and with learning (black) under H_local_. Same format as (**B**). **F** Change in trial-averaged activity for PMd and M1 under H_local_. **G** Change in covariance following adaptation. Data in **E**–**G** are presented as in **B**–**D**.

We first asked the network to learn larger VRs of 60° and 90° instead of the original 30° rotation (Fig. 7A). The model was able to compensate for these larger perturbations under both H_input_ and H_local_ (Fig. 7B, E). As expected, larger perturbations led to changes in network activity and covariance that increased with rotation angle (Fig. 7B, C, F, G). For the 90° rotation, we found a clear difference between H_input_ and H_local_: H_input_ produced larger activity changes in PMd compared to M1, opposite that under H_local_ (Fig. 7C, F). Larger rotation angles also increased the learning-related difference in covariance between H_input_ and H_local_. Under H_input_, the increase in covariance was similar for the PMd and M1 modules as the rotation increased (Fig. 7D). In contrast, under H_local_, the M1 covariance changed more with increasing rotation angle than did that of PMd (Fig. 7G). These model predictions could help differentiate between H_input_ and H_local_ in future experiments. In fact, preliminary M1 population recordings obtained during larger VRs (45° and 60°) seemed to match the model predictions for the covariance change under H_input_ (Fig. 7D stars), but not H_local_ (Fig. 7G stars).

### A visuomotor reassociation task can differentiate between H_local_ and learning through remapping of input signals

**Fig. 8:**
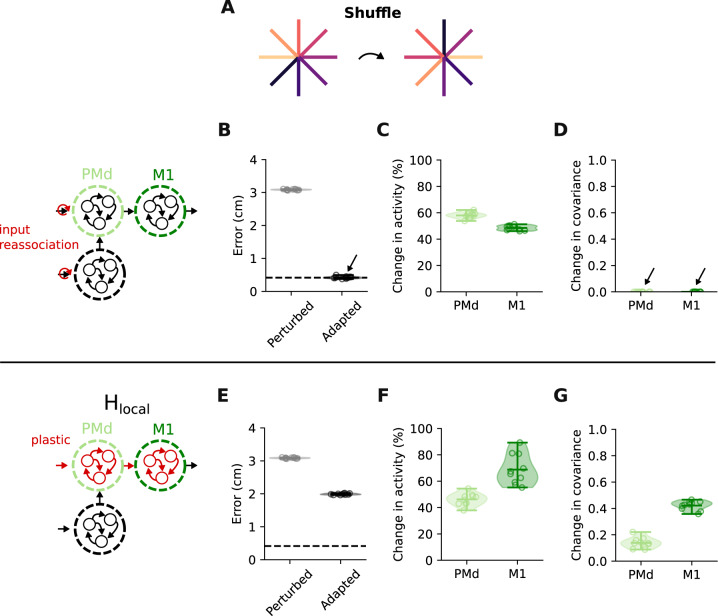
A visuomotor reassociation task can clearly differentiate between H_local_ and learning through reassociation of input signals. **A** We simulated a task in which the network had to learn new associations between target locations and reach directions (“Reassociation''). **B** Root mean squared error between target and produced hand trajectories without (grey) and with learning (black) through input reassociation. Dashed line indicates error during baseline trial. Shaded area and horizontal bars, data distribution with mean and extrema (*n* = 10 network initializations). Same data presentation in all panels. **C** Change in trial-averaged activity for PMd and M1 under input reassociation. **D** Change in covariance following adaptation. Note that the covariance matrices do not change at all. **E** Root mean squared error between target and produced hand trajectories without (grey) and with learning (black) under H_local_. **F** Change in trial-averaged activity for PMd and M1 under H_local_. **G** Change in covariance following adaptation. Data in **E**–**G** are presented as in **B**–**D**.

Although larger visuomotor rotations help differentiate between upstream learning and learning within PMd and M1, we sought to identify a task that would lead to an even clearer distinction. To this end, we implemented a reassociation task where the model had to learn a new, random mapping between cues and reaching directions (Fig. 8A; “Methods”). This task allowed us to test a very specific change in the input signal to the motor cortices that could implement adaptation^20,41^: instead of adjusting the connectivity in an upstream network (H_input_), which allows for highly unconstrained modulation of input signals, the target-related input signals were manually reordered to compensate for the reassociation of cue-reaching direction pairs (Fig. 8B). This “learning through input reassociation” resulted in large changes in network activity (Fig. 8C), comparable in magnitude to those under H_local_ (Fig. 8F). Nevertheless, it did not cause any change in covariance (Fig. 8D), which clearly distinguished it from H_local_ (Fig. 8G) and the standard H_input_ (Supplementary Fig. 8). This was the case because, in contrast to the standard H_input_ during VR adaptation, the input signals did not change per se, but were only reassigned to different targets, thereby entirely preserving the network activity patterns.

## Discussion

Rapid motor learning is associated with neural activity changes in the motor cortices. The origin of these changes remains elusive, due to the current challenge of measuring synaptic strength in vivo. Here, we have used modular RNNs to simulate the motor cortices and to explore whether learning to counteract a visuomotor rotation within tens of minutes could be mediated by local synaptic changes (H_local_). By comparing the modelled network activity changes under H_local_ to the modelled changes observed during learning upstream of the motor cortices (H_input_), we have shown how the two hypotheses could be distinguished based on neural population recordings during behaviour. Critically, despite the intuition that learning through plastic changes should lead to detectable changes in neural interactions within and across PMd and M1 populations, both H_local_ and H_input_ (Fig. 4) largely preserved the covariance within these two regions, closely matching experimental observations (Fig. 2). This likely happened because adaptation under H_local_ was achieved through small, coordinated weight changes within the PMd and M1 network modules (Fig. 5). Finally, using our model, we propose tasks for which we anticipate a more dramatic difference between these contrasting hypotheses (Figs. 7, 8) which can potentially help to interpret experimental data in the future.

Electrophysiological^10,13–15^ and modelling studies^18^, as well as psychophysical evidence^1,42^ suggest that VR adaptation is driven by areas upstream of the motor cortices. Neurophysiological evidence is largely based on the observation that the statistical interactions within PMd and M1 populations remain preserved throughout adaptation^10^. This conclusion is in good agreement with studies showing that learning to generate neural activity patterns that preserve the covariance structure only takes a few tens of minutes^19^. Our direct comparison between H_input_ and H_local_ lends further support to this observation. However, it also paints the intriguing picture that small, globally organized changes in synaptic weights could enable rapid learning without changing the neural covariance, a result that was robust across model initializations (Fig. 4), parameter settings (Supplementary Fig. 9 and Supplementary Fig. 10), architectural design choices (Supplementary Fig. 11) and learning algorithms (Supplementary Fig. 12). Even implementing the modular RNN as a spiking neural network, bringing it closer to biology, did not change this result (Supplementary Fig. 13). Our simulations thus robustly show that covariance stability is not as directly linked to stable local connectivity as previously thought, as changes in covariance were comparable between H_input_ and H_local_ for a 30° VR perturbation (Fig. 4). Instead, the change in neural covariance seemed to be more related to the task itself, as it correlated with the size of the perturbation: the larger the initial error (e.g., caused by larger rotations), the larger the change in covariance (Fig. 7). However, the relation between initial error and change in covariance differed depending on where the learning happened (H_input_ or H_local_).

The main difference between the two learning hypotheses we have examined is where in the hierarchical RNN model the connectivity changes occur: within the motor cortices (H_local_), or upstream of them (H_input_). Although neural covariance was preserved similarly by H_local_ and H_input_, we found a key characteristic that distinguished the two. When local connectivity was allowed to be plastic, the largest activity changes happened within the M1 module, with only small changes in the PMd module (Fig. 4E). In contrast, when learning occurred upstream of the PMd and M1 modules, the activity changes were similar in PMd and M1 (Fig. 4B), even if some learning was also allowed within PMd and M1 (Supplementary Fig. 10 and Supplementary Fig. 14). The experimental data, with larger activity changes in PMd than M1, better matched the pattern produced by H_input_. This observation further supports the hypothesis that VR adaptation is mediated by plasticity upstream of the motor cortices.

A more arbitrary visuomotor reassociation task allowed us to test an alternate way in which upstream learning could occur, with constraints against input signals changing but simply being reassigned to different targets (Fig. 8). Comparing this learning to that mediated by local connectivity changes revealed a clear distinction: learning under H_local_ modified the covariance in both PMd and M1, whereas learning through input reassociation preserved it^20^. Thus, future experiments seeking to disentangle to which extent learning happens within the motor cortices and/or upstream could study this task.

Studies of learning in RNNs have focused on how networks implement *de-novo* training^23,24,27,36,43–49^. However, our brain does not learn to perform any task from scratch; it has been “trained” over many generations throughout evolution^50^. Here we studied how neural networks adapt a learned behaviour, as opposed to *de-novo* learning. Our work raises the intriguing possibility that rapid learning following a few tens of minutes of practice could be easily achieved through small but specific changes in circuit connectivity. Thus, initial training seems to provide a highly flexible backbone to adapt behaviour as needed^51^.

The fact that the connectivity changes during adaptation under both H_local_ and H_input_ were small and low-dimensional (Figs. 5, 6) suggests that either one could mediate rapid learning. First, as every synaptic change is costly^52^, we would expect a constraint on the total amount of connectivity change in the brain. The VR task being solved with only minor weight changes reflects this; in fact, they could be well achieved through long-term potentiation or depression of existing synapses, as experiments have shown that synaptic strength can double within minutes^32^. Second, the low dimensionality of these weight changes is also important with respect to solving “credit assignment”, the problem of determining how each synapse should change in order to restore the desired behaviour^53–56^. Although it is still unclear how this is achieved in the brain, one possibility is that synaptic plasticity is guided by “teacher” signals^57,58^. Since neuromodulatory signals can regulate synaptic plasticity^59,60^, they seem ideal candidates to regulate biologically plausible learning^41,61–63^. The finding that the connectivity changes needed to adapt to the VR perturbation are “naturally” low-dimensional is promising, as it suggests that learning could be controlled through relatively few neuromodulatory signals. Such implementation would contrast dramatically with the daunting challenge of learning to regulate every single synapse independently. Lastly, the robustness against synaptic fluctuations conveyed by the low-dimensional connectivity changes makes both H_local_ and H_input_ attractive in terms of ensuring memory stability. Given the fluctuating nature of brain connectivity^37^, it remains puzzling how animals remember anything^38–40,64^. That low-dimensional weight changes, much smaller than ongoing synaptic fluctuations, can achieve successful behavioural adaptation provides a potential solution to this problem.

Our model consistently underestimated the changes in trial-averaged activity observed during VR adaptation, despite closely matching the small covariance changes (Fig. 2,4). This is to be expected, as the model only captures changes due to the motor adaptation process itself, whereas the actual neural activity contains signals related to other processes such as “impulsivity”^65^ or “engagement”^66^. In fact, the experimentally observed neural activity changes between the early and late trials of control reaching sessions with no perturbation were almost as large as the changes during adaptation in our model (Fig. 2C, black dots). How these changes that are not related to learning are combined with the learning-related changes studied here remains unclear. Our modelling predictions for the learning-related changes could help tackle this question in future studies.

A second potential reason why our model consistently underestimated the activity changes during adaptation could be the fact that we did not include visual or proprioceptive feedback signals in our modelling approach^67–71^. As those signals also change during adaptation, they might cause additional changes in trial-averaged neural activity, despite not being directly necessary to solve the motor adaptation task. This could explain why our model could solve the task with smaller changes in neural activity. On the other hand, feedback signals could also actively contribute to the adaptation process. From this view, we may presently overestimate the already small connectivity changes underlying VR adaptation (Fig. 5), as part of the learning process could have been instead driven by dynamic feedback signals. Thus, when taking feedback into account, rapid learning of a motor perturbation could potentially be realized with even smaller changes in underlying connectivity, or maybe even without any connectivity changes at all^2^. To this point, the concrete role of feedback for rapid motor learning remains unclear and it could be interesting to use our model to further investigate this question.

Our simulations were not designed to study trial-by-trial learning: we were interested in the neural activity changes between the baseline and late adaptation phases when the subjects had largely learned to counteract the perturbation and reached stable behaviour (Fig. 2B). Given that motor adaptation seems to be mediated by two processes with different timescales^72–74^, our model mainly captures the slower of the two. The neural activity changes underlying the early phase adaptation may be driven by different processes^10^, which our model currently does not test.

In conclusion, our comparison between the activity changes following VR adaptation through plastic changes within or upstream of the motor cortices shows that local plasticity (H_local_) leads to neural signatures that are unexpectedly similar to those of upstream learning (H_input_). Intriguingly, H_local_ not only largely preserved the covariance within PMd and M1 but also resulted in connectivity changes that seem biologically reasonable: they are small, make the network robust against synaptic fluctuations, and can be controlled by relatively few teaching signals. Our simulations thus encourage caution when drawing conclusions from the analysis of neural population recordings during learning, and further suggest potential behavioural tasks that could make it easier to identify where learning is happening within the motor system.

## Methods

### Tasks

We studied motor adaptation using a visuomotor rotation (VR) paradigm, previously described in Perich et al. 2018^10^. Monkeys (*macaca mulatta*) performed an instructed delay centre-out-reaching task in which they had to reach to one of eight targets uniformly distributed along a circle. All targets were 2 cm squares. The delay period was variable and ranged between 500 and 1500 ms. For additional details on the task, see^10^. During the adaptation phase, visual feedback was rotated clockwise or counterclockwise by 30°, 45°, or 60°. All surgical and experimental procedures were approved by the Institutional Animal Care and Use Committee (IACUC) of Northwestern University. Using our modular RNN model, we simulated both this task and a visuomotor reassociation task in which there was no consistent rotation of the visual feedback; instead, each target required reaching to a different direction, uniquely selected from the initial set of eight different targets.

### Experimental recordings

We analyzed eleven sessions from two monkeys (five for Monkey C, six for Monkey M) that were exposed to a clockwise or counterclockwise 30° rotation (data previously presented in^10^). In addition to these data, we also analyzed three control sessions (one for Monkey C, two for Monkey M) in which no perturbation was applied, as well as additional sessions with larger VR angles from Monkey C where only M1 data was collected (30°, nine sessions; 45°, two sessions; 60°, two sessions) (Fig. 7).

The spiking activity of putative single neurons was binned into 10 ms bins and then smoothed using a Gaussian filter (s.d., 50 ms). Only successful trials, where monkeys received a reward at the end, were included in the analysis. We defined the early and late adaptation epochs as the first and last 150 trials of the perturbation phase, when the visuomotor rotation was applied, respectively.

### RNN model

#### Architecture

The neural network contained three recurrent modules, each consisting of 400 neurons, which we refer to as upstream, PMd and M1, respectively (Fig. 3A). The PMd and the upstream modules received an identical three-dimensional input signal, with the first two dimensions signalling the *x* and *y* target location of that trial, and the third dimension signalling go (1 until the go, and 0 from then on). The upstream module connects to the PMd module and the PMd module connects to the M1 module. The output is calculated as a linear readout of the M1 module activity. Recurrent, as well as feedforward connections were all-to-all. The model dynamics are given by

1 $${{{{{{{{\bf{x}}}}}}}}}_{t+1}^{{{{{\rm{UP}}}}}}={{{{{{{{\bf{x}}}}}}}}}_{t}^{{{{{\rm{UP}}}}}}+\frac{dt}{\tau }\left(-{{{{{{{{\bf{x}}}}}}}}}_{t}^{{{{{\rm{UP}}}}}}+{{{{{{{{\bf{W}}}}}}}}}^{{{{{\rm{UP}}}}}}\tanh ({{{{{{{{\bf{x}}}}}}}}}_{t}^{{{{{\rm{UP}}}}}})+{{{{{{{{\bf{W}}}}}}}}}^{{{{{{\rm{in}}}}}},{{{{{\rm{UP}}}}}}}{{{{{{{{\bf{s}}}}}}}}}_{t}\right)$$

2 $${{{{{{{{\bf{x}}}}}}}}}_{t+1}^{{{{{\rm{PMd}}}}}}={{{{{{{{\bf{x}}}}}}}}}_{t}^{{{{{\rm{PMd}}}}}}+\frac{dt}{\tau }\left(-{{{{{{{{\bf{x}}}}}}}}}_{t}^{{{{{\rm{PMd}}}}}}+{{{{{{{{\bf{W}}}}}}}}}^{{{{{\rm{PMd}}}}}}\tanh ({{{{{{{{\bf{x}}}}}}}}}_{t}^{{{{{\rm{PMd}}}}}})+{{{{{{{{\bf{W}}}}}}}}}^{{{{{{\rm{UP}}}}}}{\mbox{-}}{{{{{\rm{PMd}}}}}}}\tanh ({{{{{{{{\bf{x}}}}}}}}}_{t}^{{{{{\rm{UP}}}}}})+{{{{{{{{\bf{W}}}}}}}}}^{{{{{{\rm{in}}}}}},{{{{{\rm{PMd}}}}}}}{{{{{{{{\bf{s}}}}}}}}}_{t}\right)$$

3 $${{{{{{{{\bf{x}}}}}}}}}_{t+1}^{{{{{\rm{M1}}}}}}={{{{{{{{\bf{x}}}}}}}}}_{t}^{{{{{\rm{M1}}}}}}+\frac{dt}{\tau }\left(-{{{{{{{{\bf{x}}}}}}}}}_{t}^{{{{{\rm{M1}}}}}}+{{{{{{{{\bf{W}}}}}}}}}^{{{{{\rm{M1}}}}}}\tanh ({{{{{{{{\bf{x}}}}}}}}}_{t}^{{{{{\rm{M1}}}}}})+{{{{{{{{\bf{W}}}}}}}}}^{{{{{{\rm{PMd}}}}}}{\mbox{-}}{{{{{\rm{M1}}}}}}}\tanh ({{{{{{{{\bf{x}}}}}}}}}_{t}^{{{{{\rm{PMd}}}}}})\right)$$

4 $${{{{{{{{\bf{x}}}}}}}}}_{t}^{{{{{\rm{out}}}}}}={{{{{{{{\bf{W}}}}}}}}}^{{{{{\rm{out}}}}}}\tanh ({{{{{{{{\bf{x}}}}}}}}}_{t}^{{{{{\rm{M1}}}}}})+{{{{{{{{\bf{b}}}}}}}}}^{{{{{\rm{out}}}}}}$$

where **x**^UP^ describes the network activity in the upstream module, and **x**^PMd^ and **x**^M1^ the network activity in the PMd and M1 module respectively. **W**^UP^, **W**^PMd^ and **W**^M1^ define the recurrent connectivity matrix within the upstream module, the PMd module and the M1 module, respectively. **W**^UP-PMd^ defines the connectivity matrix from the upstream module to the PMd module, and **W**^PMd-M1^ defines the connectivity matrix from the PMd module to the M1 module. The input connectivity matrices for the upstream and the PMd module are given by **W**^in,UP^ and **W**^in,PMd^, respectively; **s**_*t*_ represents the three-dimensional input signal described above. The two-dimensional output **x**^out^ is decoded from the M1 module activity via the output connectivity matrix **W**^out^ and the bias term **b**^out^. The time constant is *τ* = 0.05 s and the integration time step is *dt* = 0.01 s.

#### Training

Each network was initially trained to produce planar reaching trajectories, mirroring the experimental hand trajectories. The training and testing data set were constructed by pooling the hand trajectories **x**^target^ for successful trials during the baseline epochs from all experimental sessions, which resulted in 2238 trials of length 4 s (90%/10% randomly split into training/testing). The held out testing data was used to validate that the model had been trained successfully during the initial training period. Model simulations were implemented using PyTorch^75^ and training was performed using the Adam optimizer^76^ with a learning rate of 0.0001 (*β*_1_ = 0.9, *β*_2_ = 0.999). The initial training consisted of 500 training trials. The loss function was defined as

5 $$L=\frac{1}{B(T-50)2}\mathop{\sum }\limits_{b}^{B}\mathop{\sum }\limits_{t =50}^{T}\mathop{\sum}\limits_{d=x,y}{\left({{{{{{{x}}}}}}}_{t,b}^{{{{{{\rm{out}}}}}},d}-{{{{{{{x}}}}}}}_{t,b}^{{{{{{\rm{target}}}}}},d}\right)}^{2}+{E}^{{{{{\rm{weights}}}}}}+{E}^{{{{{{\rm{rates}}}}}}}$$

where the regularization term on the weights is given by (∣∣ . ∣∣ indicates L2 norm)

6 $${E}^{{{{{\rm{weights}}}}}}=	\alpha (|\vert {{{{{{{{\bf{W}}}}}}}}}^{{{{{\rm{in,UP}}}}}}|\vert+\vert|{{{{{{{{\bf{W}}}}}}}}}^{{{{{\rm{in,PMd}}}}}}|\vert+\vert|{{{{{{{{\bf{W}}}}}}}}}^{{{{{\rm{out}}}}}}|\vert+\vert|{{{{{{{{\bf{W}}}}}}}}}^{{{{{\rm{PMd}}}}}}|\vert+\vert|{{{{{{{{\bf{W}}}}}}}}}^{{{{{\rm{M1}}}}}}|\vert \\ 	+|\vert {{{{{{{{\bf{W}}}}}}}}}^{{{{{{\rm{PMd}}}}}}{\mbox{-}}{{{{{\rm{M1}}}}}}}|\vert+\vert|{{{{{{{{\bf{W}}}}}}}}}^{{{{{\rm{UP}}}}}}|\vert+\vert|{{{{{{{{\bf{W}}}}}}}}}^{{{{{{\rm{UP}}}}}}{\mbox{-}}{{{{{\rm{PMd}}}}}}}|\vert )$$

the regularization term on the rates is given by

7 $${E}^{{{{{\rm{rates}}}}}}=\beta \frac{1}{BTN}\mathop{\sum }\limits_{b}^{B}\mathop{\sum }\limits_{t}^{T}\mathop{\sum }\limits_{n}^{N}\left({\left(\tanh \left({{{{{{{x}}}}}}}_{t,b}^{{{{{{\rm{PMd}}}}}},n}\right)\right)}^{2}+{\left(\tanh \left({{{{{{{x}}}}}}}_{t,b}^{{{{{{\rm{M1}}}}}},n}\right)\right)}^{2}+{\left(\tanh \left({{{{{{{x}}}}}}}_{t,b}^{{{{{{\rm{UP}}}}}},n}\right)\right)}^{2}\right)$$

with batch size *B* = 80, time steps *T* = 400 and neurons *N* = 400. The regularization parameters were set to *α* = 0.001, *β* = 0.8. We clipped the gradient norm at 0.2 before we applied the optimization step. For the VR adaptation, we trained the initial network for another 100 trials with the target trajectory rotated 30° (or 60° or 90° for the case of the larger VRs). For the VR reassociation task we shuffled the stimulus **s** across reaching directions but kept the targets **x**^target^ fixed, as indicated in Fig. 8A (colours correspond to the given stimulus and sketched reaching trajectories correspond to the assigned target). The network had again 100 trials to adapt to this perturbation.

### Data analysis

We quantified the changes in actual and simulated neural activity following adaptation using two measures: changes in trial-averaged activity (or peristimulus time histogram, PSTH), and changes in covariance. We calculated both metrics within a window that started 600 ms before the go signal and ended 600 ms after it. The change in activity was calculated by

8 $$\frac{|{{{{{\rm{PSTH}}}}}}^{{{{{\rm{Lateadaptation}}}}}}-{{{{{\rm{PSTH}}}}}}^{{{{{\rm{Baseline}}}}}}|}{{\sigma }^{{{{{\rm{Baseline}}}}}}}$$

where PSTH^Baseline^ is the trial-averaged activity in the baseline epoch (experimental data: all baseline trials; simulated data: on a trained model, 100 trials with similar go signal timing), PSTH^Lateadaptation^ is the trial-averaged activity in the late adaptation epoch (experimental data: last 150 trials of the adaptation epoch; simulation data: on a model trained to counteract the perturbation, 100 trials with similar go signal timing), and *σ*^Baseline^ is the neuron-specific standard deviation across time and targets during the baseline epoch. To summarize the change in trial averaged activity across all neurons, time points, and targets, we calculated their median; this provided one single value for each experimental session or simulation run. The change in covariance was calculated using the same trial-averaged data from the baseline and the late adaptation epoch. We calculated the covariance in each of these two epochs and then quantified the similarity by calculating the Pearson correlation coefficient between the corresponding entries of the two matrices. The change in covariance is then defined by 1 minus the correlation coefficient. For the experimental sessions, we computed a lower bound for each measure using the control sessions in which monkeys were not exposed to a perturbation. To account for the fact that there could be activity changes unrelated to motor adaptation^65,66^, we compared the activity during 150 consecutive trials from the first half of the control session with 150 consecutive trials from the second half of the control session.

To compute the magnitude of the weight changes after networks learned to counteract the perturbation, we computed the average absolute weight change as

9 $${{{{{{{\bf{dW}}}}}}}}=\frac{|{{{{{{{{\bf{W}}}}}}}}}^{{{{{\rm{Lateadaptation}}}}}}-{{{{{{{{\bf{W}}}}}}}}}^{{{{{\rm{Baseline}}}}}}|}{{{{{{{{{\bf{W}}}}}}}}}^{{{{{\rm{Baseline}}}}}}}$$

where ∣ . ∣ indicates the element wise absolute value, **W**^Baseline^ is defined as the model parameter (either **W**^in,PMd^, **W**^in,UP^, **W**^UP^, **W**^UP-PMd^, **W**^PMd^, **W**^PMd-M1^, or **W**^M1^) after the initial training phase but before training on the VR perturbation, and **W**^Lateadaptation^ is defined as the same model parameter after training on the VR perturbation. To obtain one summary value for each simulation run, we calculated the median of all weight entries for a given parameter. To measure the dimensionality of weight change we calculated the singular values *k*_*i*_ of **W**^Lateadaptation^ − **W**^Baseline^ and defined the dimensionality, using the participation ratio^77^:

10 $${\left(\mathop{\sum}\limits_{i}{k}_{i}\right)}^{2}/\mathop{\sum}\limits_{i}{k}_{i}^{2}$$

### Statistics

To statistically compare the change in activity found in the control sessions with the change found in the VR sessions, we performed a linear mixed model analysis using R (lmer package). The brain area (PMd or M1) and whether the experimental session included a perturbation phase or not were included as fixed effects, whereas monkey and session identity were included as random effects. A significance threshold of *P* = 0.05 was used.

### Simulation of synaptic fluctuation (Fig. 6)

To simulate synaptic fluctuation we added random values to the learned connectivity changes during adaptation. Those random values were drawn from a normal distribution with zero mean and s.d. ten times larger than the s.d. of the learned weight changes distribution. With that, we created synaptic noise which was completely unstructured across connection sites. We did not add or delete any synapses in the model.

### Reporting summary

Further information on research design is available in the Nature Research Reporting Summary linked to this article.

## Supplementary information

Supplementary Information

Reporting Summary

## Data Availability

The data that support the findings in this study are available from the corresponding authors upon reasonable request. Source data are provided with this paper.

## Source data

Source Data

## Code availability

All code to reproduce the main simulation results can be found on GitHub (https://github.com/babaf/motor-adaptation-local-vs-input.git).
