# Supplementary material for: Small, correlated changes in synaptic connectivity may facilitate rapid motor learning
Source: Nat Commun. 2022 Sep 2;13:5163. doi: 10.1038/s41467-022-32646-w (PMC9440011; doi:10.1038/s41467-022-32646-w)
Supplement: Supplementary file 1 — Supplementary Information [file 41467_2022_32646_MOESM1_ESM.pdf]

Supplementary Figures

Supplementary Figure 1. Model kinematics closely match mean target trajectories.

Supplementary Figure 2. Model activity resembles neural data in an area-specific fashion.

Supplementary Figure 3. Actual and simulated activity changes within the neural manifold.

Supplementary Figure 4. Analysis of weight changes following VR adaptation under H_{input}.

Supplementary Figure 5. Analysis of weight changes following VR adaptation under H_{local}.

Supplementary Figure 6. Weight changes after initial training.

Supplementary Figure 7. Sensitivity of change in activity and covariance to magnitude of weight change.

Supplementary Figure 8. Adaptation to a visuomotor reassociation perturbation under H_{input}.

Supplementary Figure 9. Results are robust to parameter variations.

Supplementary Figure 10. Results are consistent for different plasticity combinations.

Supplementary Figure 11. Results are robust to variations in model architecture.

Supplementary Figure 12. Results are robust to variations in training algorithm.

Supplementary Figure 13. A modular spiking neural network replicates the main results obtained using a rate-based network.

Supplementary Figure 14. Analysis of weight changes for H_{all}.

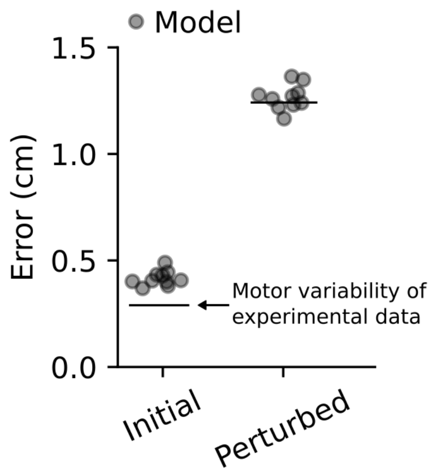

Supplementary Figure 1: Model kinematics closely match mean target trajectories. Root mean squared error (RMSE) between target and simulated hand trajectories (circles) after initial training (left) and after a VR perturbation is introduced (right). To test where the remaining error after initial training is coming from, we calculated the error relating to the motor variability inherent in the experimental data. For this, we calculated the average RMSE between individual and trial-averaged monkey hand trajectories (lines), resulting in an error close to our model's performance (circles).

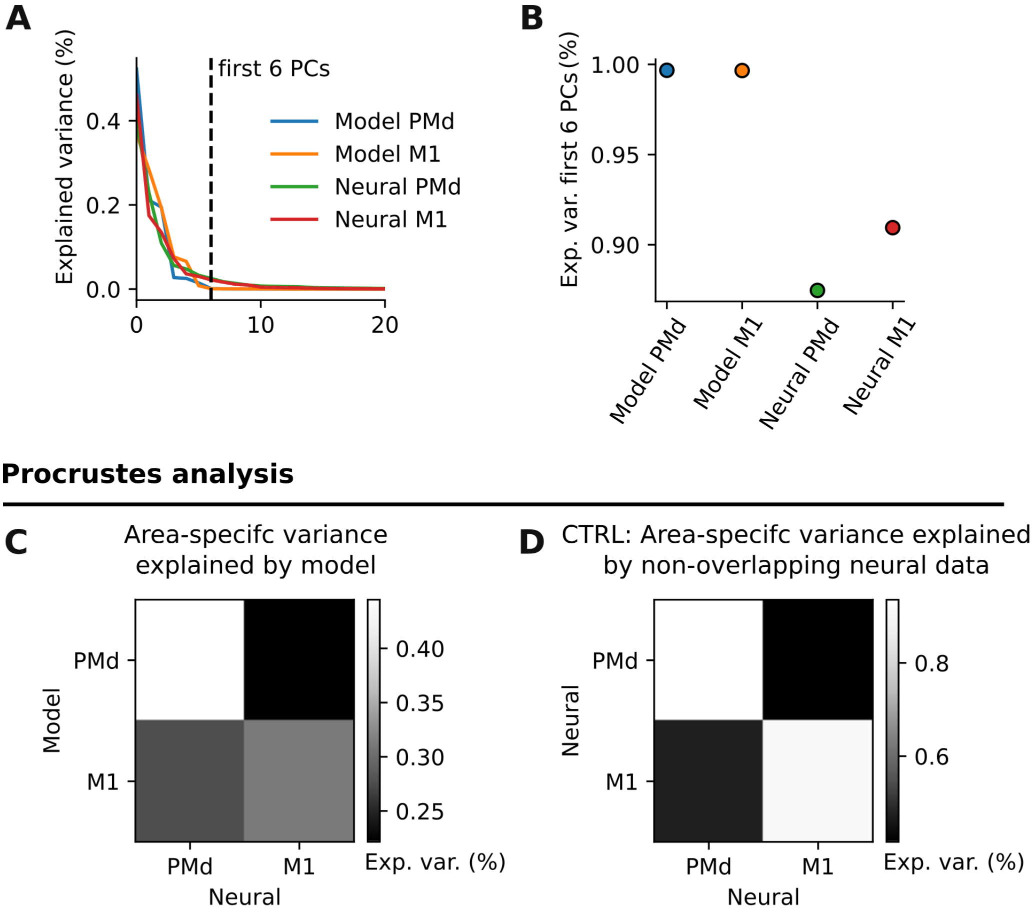

Supplementary Figure 2: Model activity resembles neural data in an area-specific fashion. **A.** Percentage of total variance explained for example model PMd (blue), model M1 (orange), monkey PMd (green) and monkey M1 (red). Principal component analysis was performed on trial-averaged activity. **B.** Total percentage of variance explained by the first six principal components for the data in A. **C.** Investigation of the area-specificity of model PMd and M1. Procrustes analysis performed on trial-averaged activity, projected onto the first six principal components. Model PMd best explained monkey PMd, and model M1 best explained monkey M1. **D.** Validation of Procrustes analysis as a measure of similarity of neural activity across different time windows: a comparison of two non-overlapping subsets of trials from monkey PMd and M1 shows that each area most resembles itself.

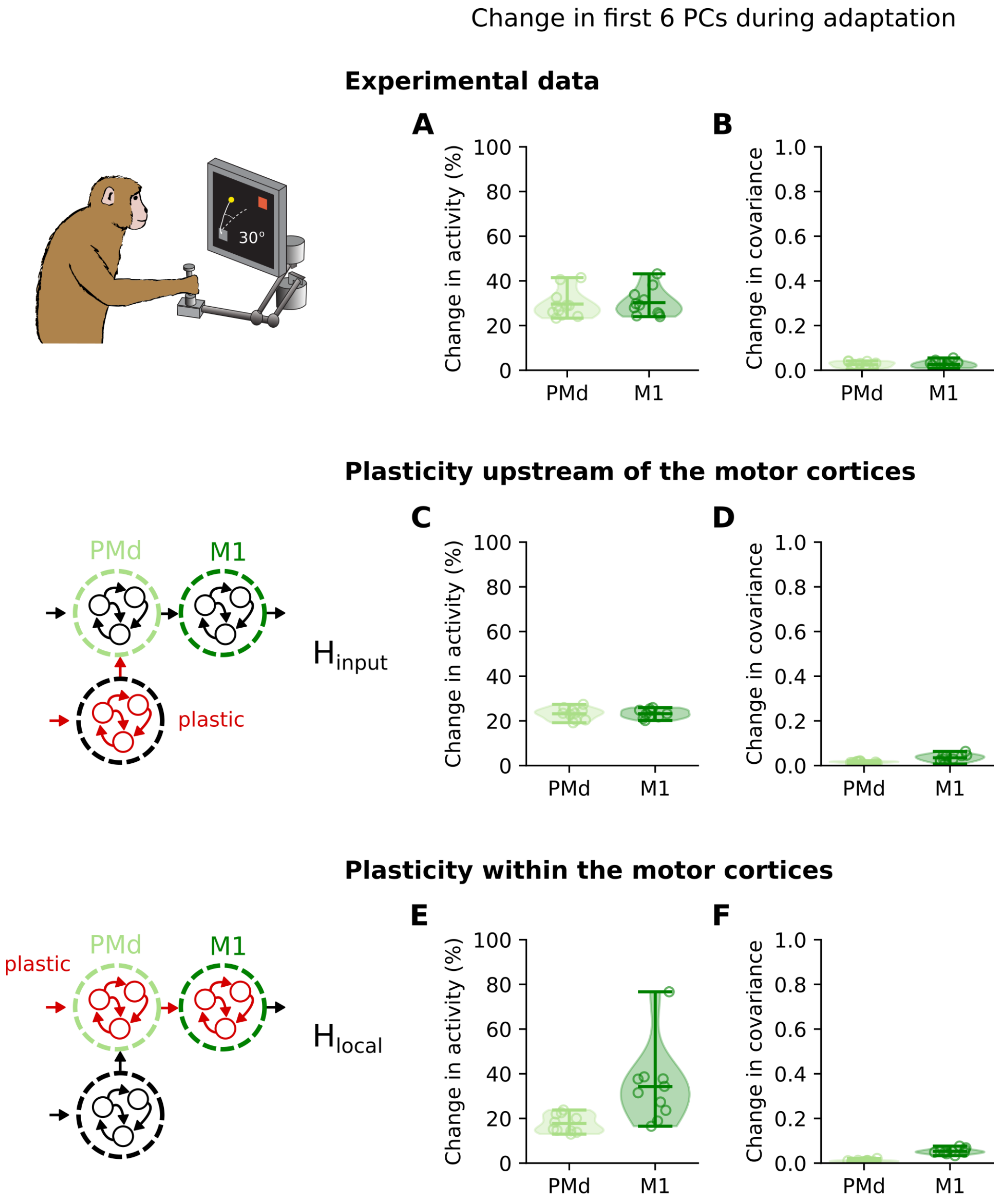

Supplementary Figure 3: Actual and simulated activity changes within the neural manifold. Here, we focused on the population-wide activity changes, rather than the single neuron/unit changes. We used Principal Component Analysis to identify a six-dimensional neural manifold that captured >80% of the trial-averaged activity (during the baseline epoch). **A.** Changes in trial-averaged activity for the PMd and M1 experimental data. Horizontal lines represent minimum, mean, maximum, empty circles individual data points (n=10 network initialisations). **B.** Change in neural covariance for each of these two regions; data presented as in A. **C.** Change in trial-averaged activity following adaptation under H_{input}. **D.** Change in covariance following adaptation under H_{input}. **E.** Change in trial-averaged activity following adaptation under H_{local}. **F.** Change in covariance following adaptation under H_{local}. Data in C,D and E,F are presented as in A and B, respectively. The monkey image was created by Carolina Massumoto who gave permission to use it under CC-BY license.

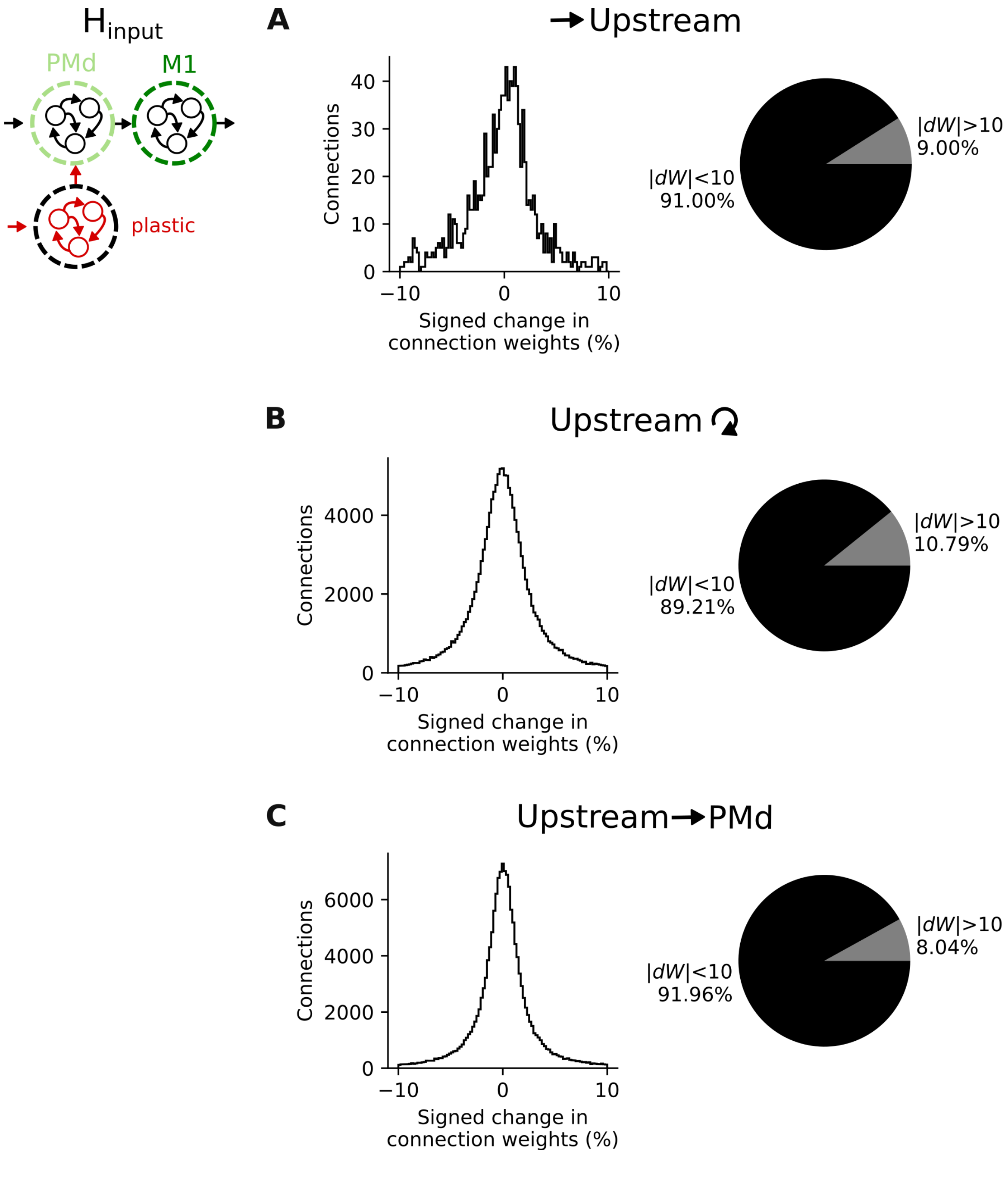

Supplementary Figure 4: Analysis of weight changes following VR adaptation under $\mathbf{H_{input}}$. A. Distribution of signed weight changes (left) and proportion of connections showing a large absolute weight change ($|\mathbf{dW}| > 10$) (right) for the input connections of the upstream network module. **B.** Weight changes for the recurrent connections of the upstream network module. **C.** Weight changes for the upstream to PMd connections. Data in B,C is presented as in A. Data of one representative network simulation is shown.

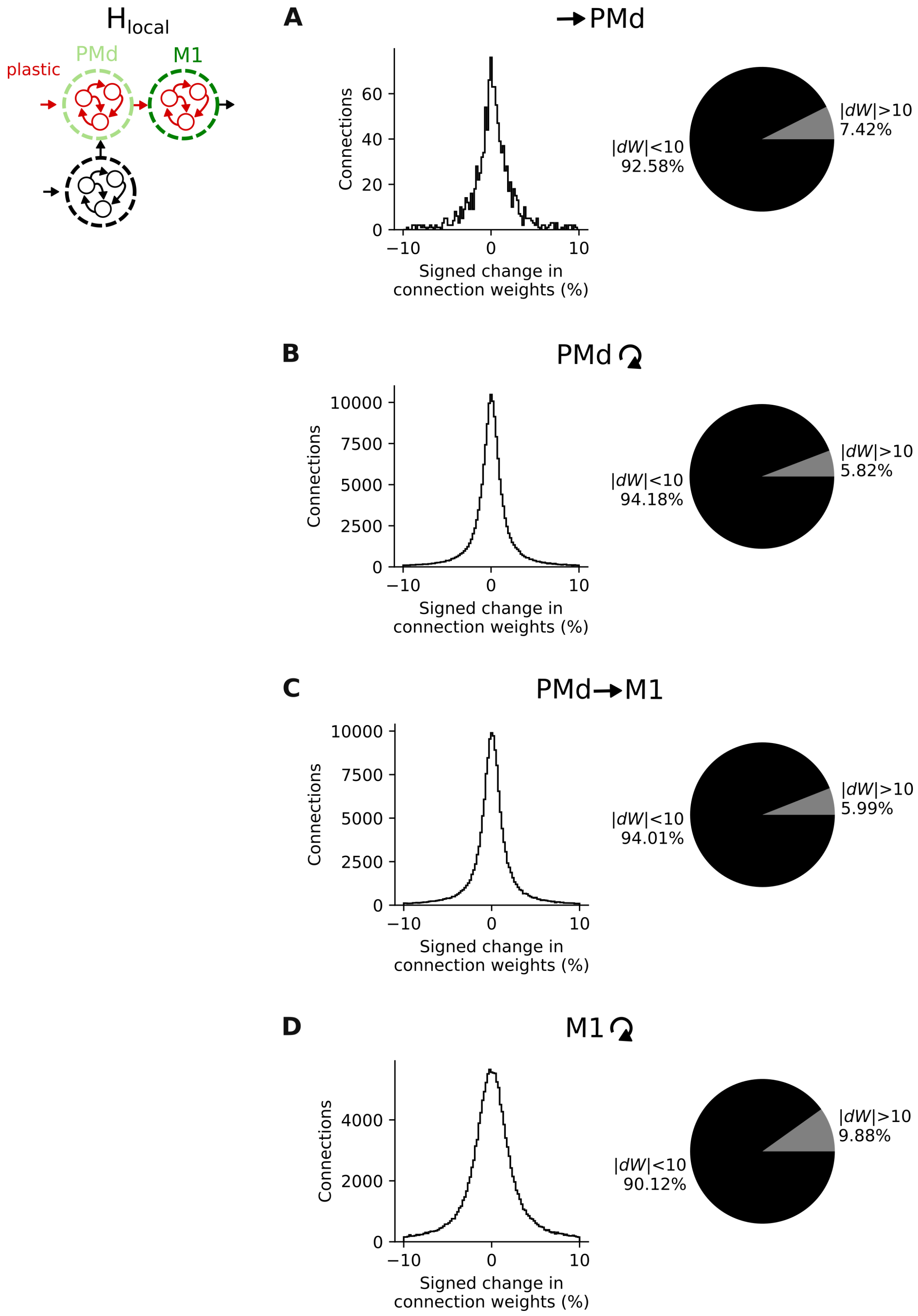

Supplementary Figure 5: Analysis of weight changes following VR adaptation under $\mathbf{H_{local}}$. A. Distribution of signed weight changes (left) and proportion of connections showing a large absolute weight change ($|\mathbf{dW}| > 10$) (right) for the input connections of the PMd network module. **B.** Weight changes for the recurrent connections of the PMd module. **C.** Weight changes for the PMd to M1 connections. D. Weight changes for the recurrent connections of the M1 module. Data in B,C,D is presented as in A. Data of one representative network simulation is shown.

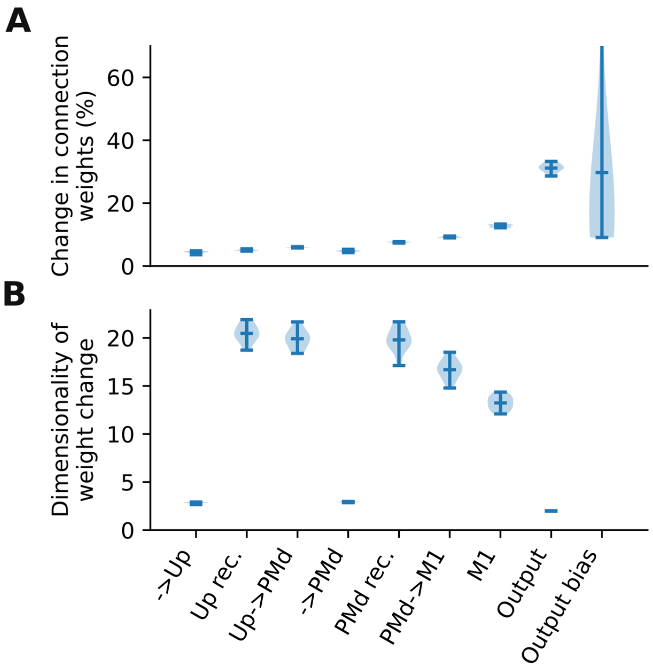

Supplementary Figure 6: Weight changes after initial training. A. Average magnitude of weight changes following initial training on the reaching task. Shaded area and horizontal bars, data distribution with mean and extrema (n=10 network initialisations). **B.** Dimensionality of weight changes following initial training on the reaching task. Data presented as in A.

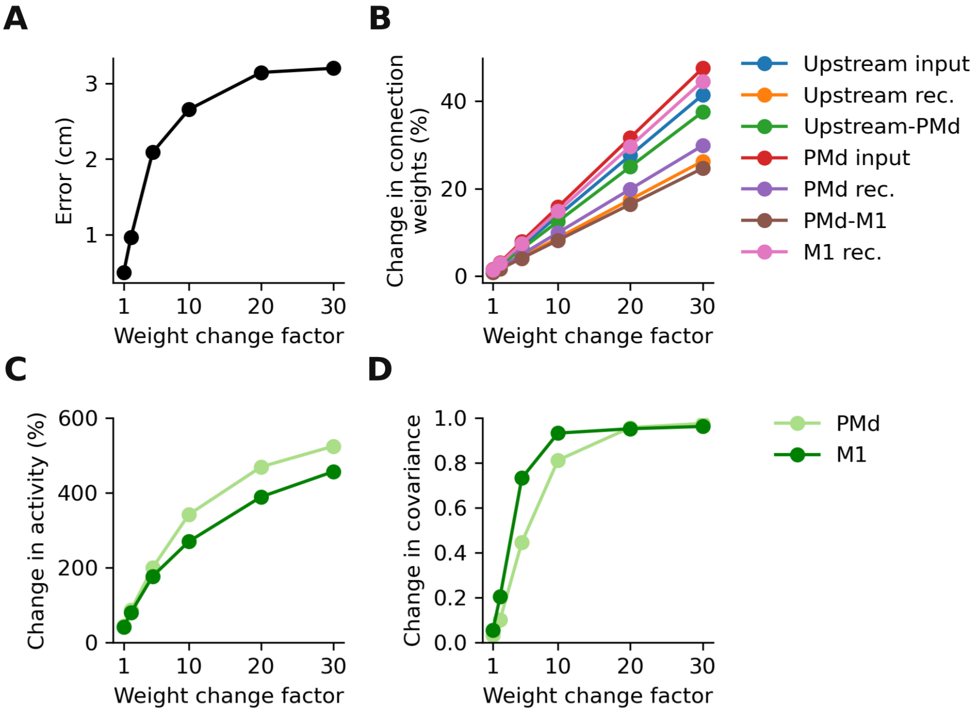

Supplementary Figure 7: Sensitivity of change in activity and covariance to magnitude of weight change. A. VR adaptation performance, measured as root mean squared error between target and produced hand trajectories. Shown is the performance after adaptation for a model where every parameter is allowed to be plastic (H_{all} in Supplementary Figure 10). To test the sensitivity of the network activity and the produced output on the magnitude of the underlying connectivity changes, we scaled up the learned weight changes during VR adaptation by a factor (x-axis). A weight change factor of 1 thus corresponds to the true weight changes observed after adaptation. Increasing the learned weight changes deteriorates performance, as error increases for increasing weight change factor. **B.** Measured weight change, shown for all model parameters. **C.** Change in activity in the PMd (light) and M1 (dark) modules, respectively. **D.** Change in covariance. Data shown as in C. C,D show that the change in activity and covariance is highly sensitive to the magnitude of the weight change.

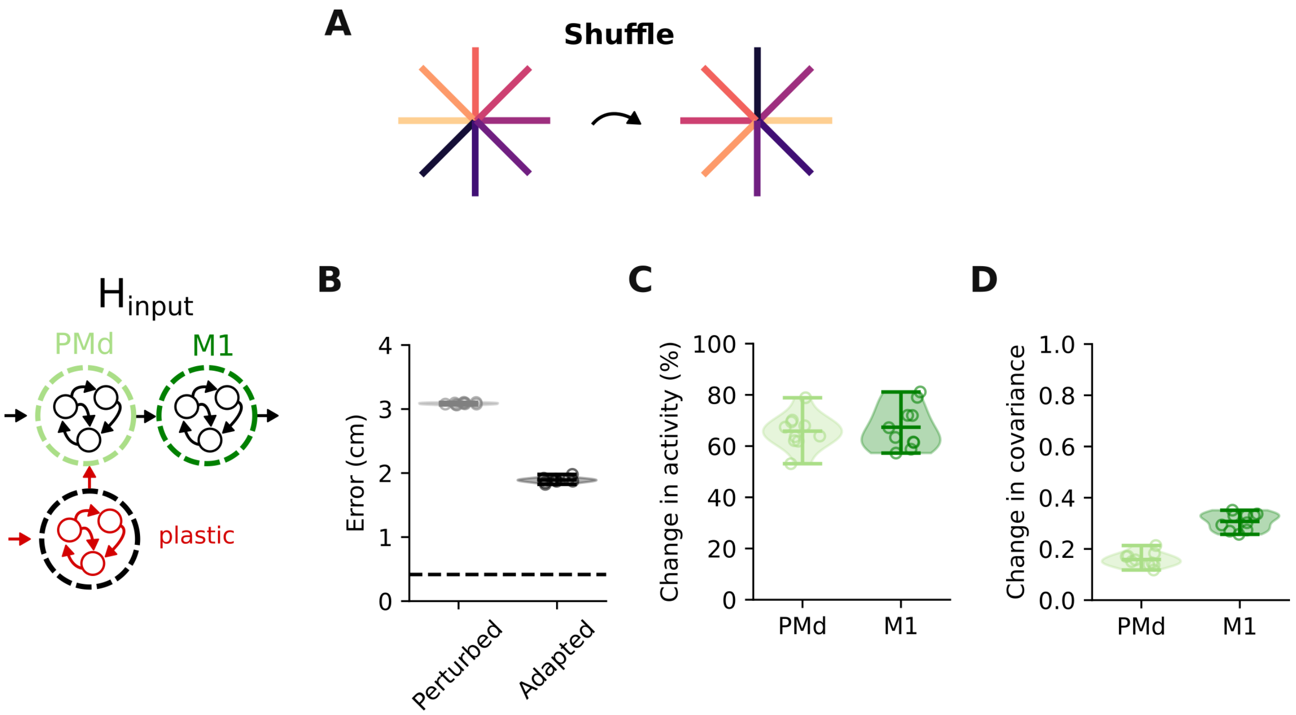

Supplementary Figure 8: Adaptation to a visuomotor reassociation perturbation under $\mathbf{H_{input}}$. **A.** We simulated a reassociation task in which the network had to learn new associations between inputs and reach directions. **B.** Root mean squared error between target and produced hand trajectories without (grey) and with learning (black) under H_{input}. Dashed line indicates error during baseline trials. Shaded area and horizontal bars, data distribution with mean and extrema (n=10 network initialisations). Same data presentation in all panels. **C.** Change in trial-averaged activity for PMd and M1. **D.** Change in covariance. Data in C,D are presented as in B.

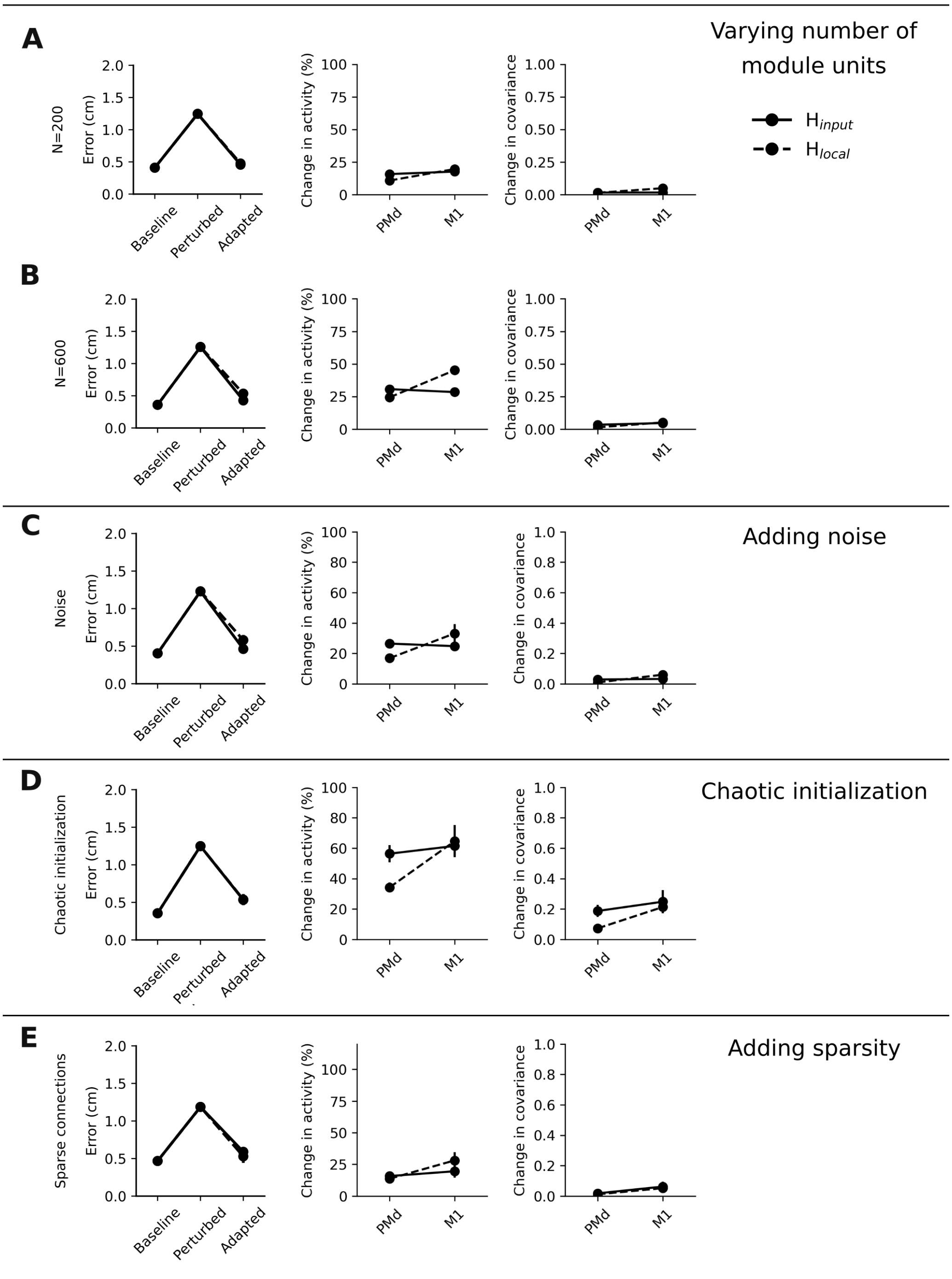

Supplementary Figure 9: Results are robust to parameter variations. A.,**B.** Models with different number of units in each network module exhibit qualitatively similar activity changes. VR adaptation performance, measured as root mean squared error between target and produced hand trajectories (left), change in activity following adaptation (middle), and change in covariance following adaptation (right). Data in B,C,D,E is presented as in A. **C.** Adding noise to the network does not fundamentally change the activity changes in the network. Each neuron in the model received an additional, random, independent input at each time step, drawn from a normal distribution with zero mean and s.d. 0.1. Markers and error bars, mean and s.d. (n=10 network initialisations). **D.** Chaotic initialization slightly increases the overall change in activity and covariance following adaptation, yet preserves the fact that H_{input} leads to larger changes in PMd compared to M1. Recurrent and inter module weights were initially drawn from a normal distribution with zero mean and s.d. $1.2/\sqrt(N)$. Markers and error bars, mean and s.d. (n=10 network initialisations). **E.** Networks with sparse recurrent and inter module connectivity show similarly low changes in activity and covariance. Only 60% of inter module and 80% of recurrent weights were allowed to be non-zero. Only non-zero connections were plastic during initial training and adaptation. Markers and error bars, mean and s.d. (n=10 network initialisations).

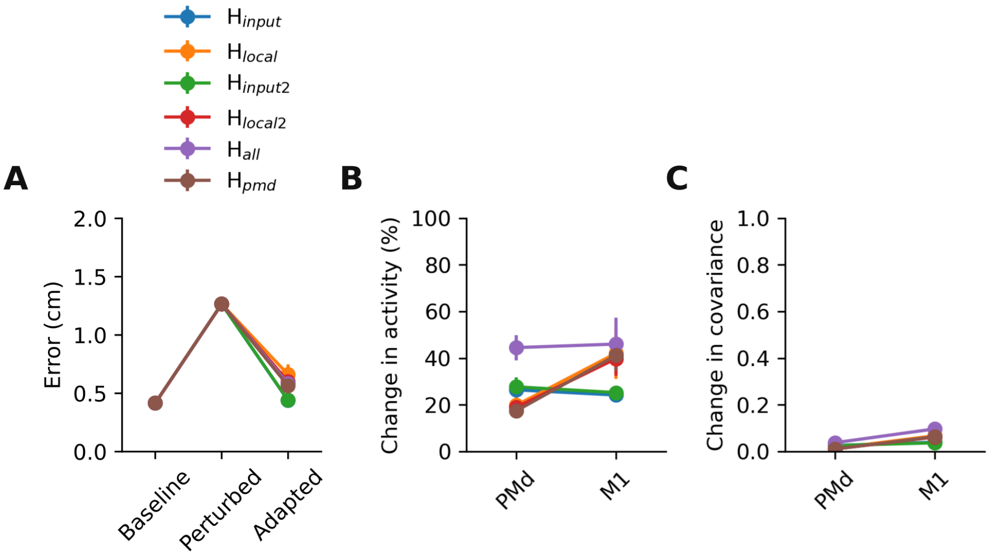

Supplementary Figure 10: Results are consistent for different plasticity combinations. **A.** VR adaptation performance, measured as root mean squared error between target and produced hand trajectories. Colours indicate which parameters of the model were allowed to be plastic during adaptation. $\mathrm{H}_{\mathrm{input2}}$ is similar to $\mathrm{H}_{\mathrm{input}}$ except that the input weight to PMd ($\mathbf{W}^{in,PMd}$) is also plastic. $\mathrm{H}_{\mathrm{local2}}$ is similar to $\mathrm{H}_{\mathrm{local}}$ except that the input weight to PMd ($\mathbf{W}^{in,PMd}$) is not plastic. For $\mathrm{H}_{\mathrm{all}}$ every parameter is plastic. For $\mathrm{H}_{\mathrm{pmd}}$ only the recurrent connectivity within PMd is plastic. Markers and error bars, mean and s.d. (n=10 network initialisations), same in all panels. **B.** Change in activity following adaptation. **C.** Change in covariance following adaptation.

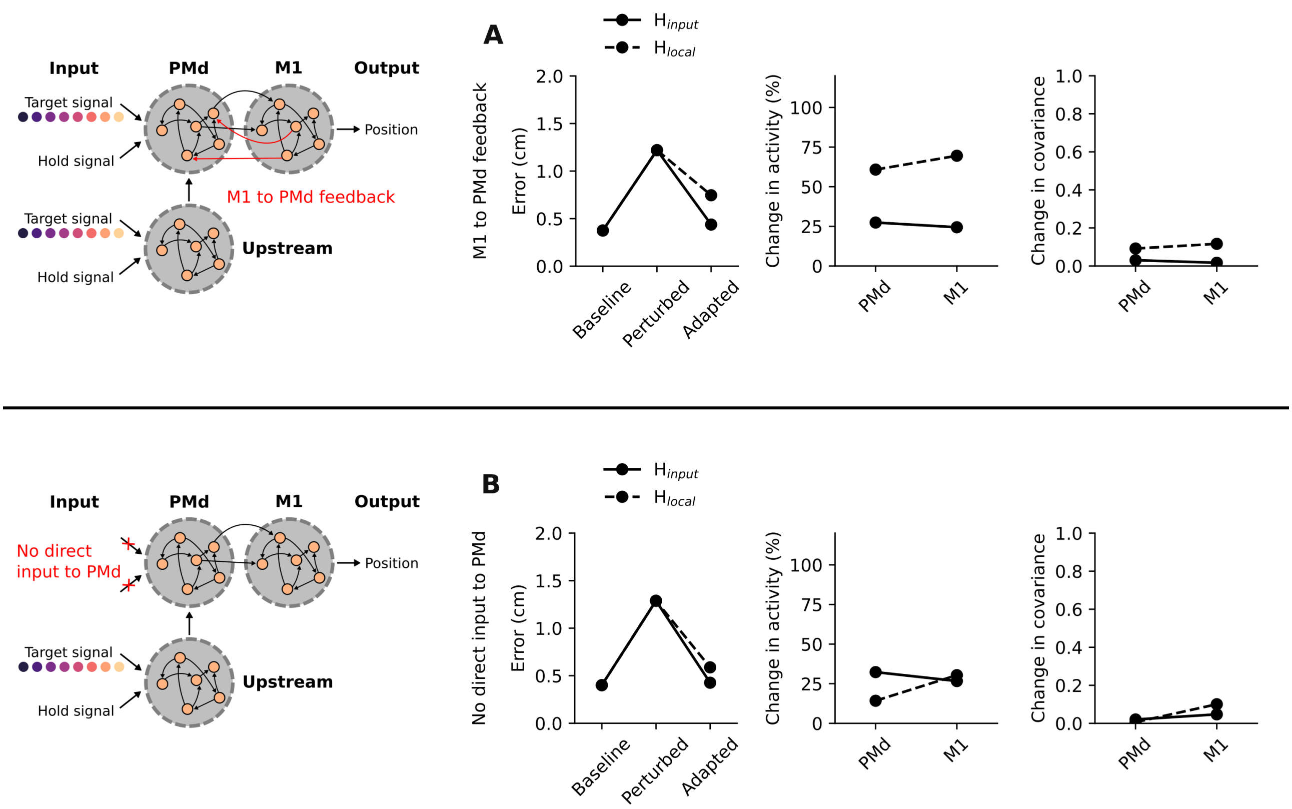

Supplementary Figure 11: Results are robust to variations in model architecture. A. Model with all-to-all feedback connections from M1 to PMd: VR adaptation performance, measured as the root mean squared error between target and produced hand trajectories (left); change in trial-averaged activity following adaptation (middle); and change in covariance following adaptation (right). Markers and error bars, mean and s.d. (n=10 network initialisations). **B.** Model with no direct input to PMd; data presented as in A.

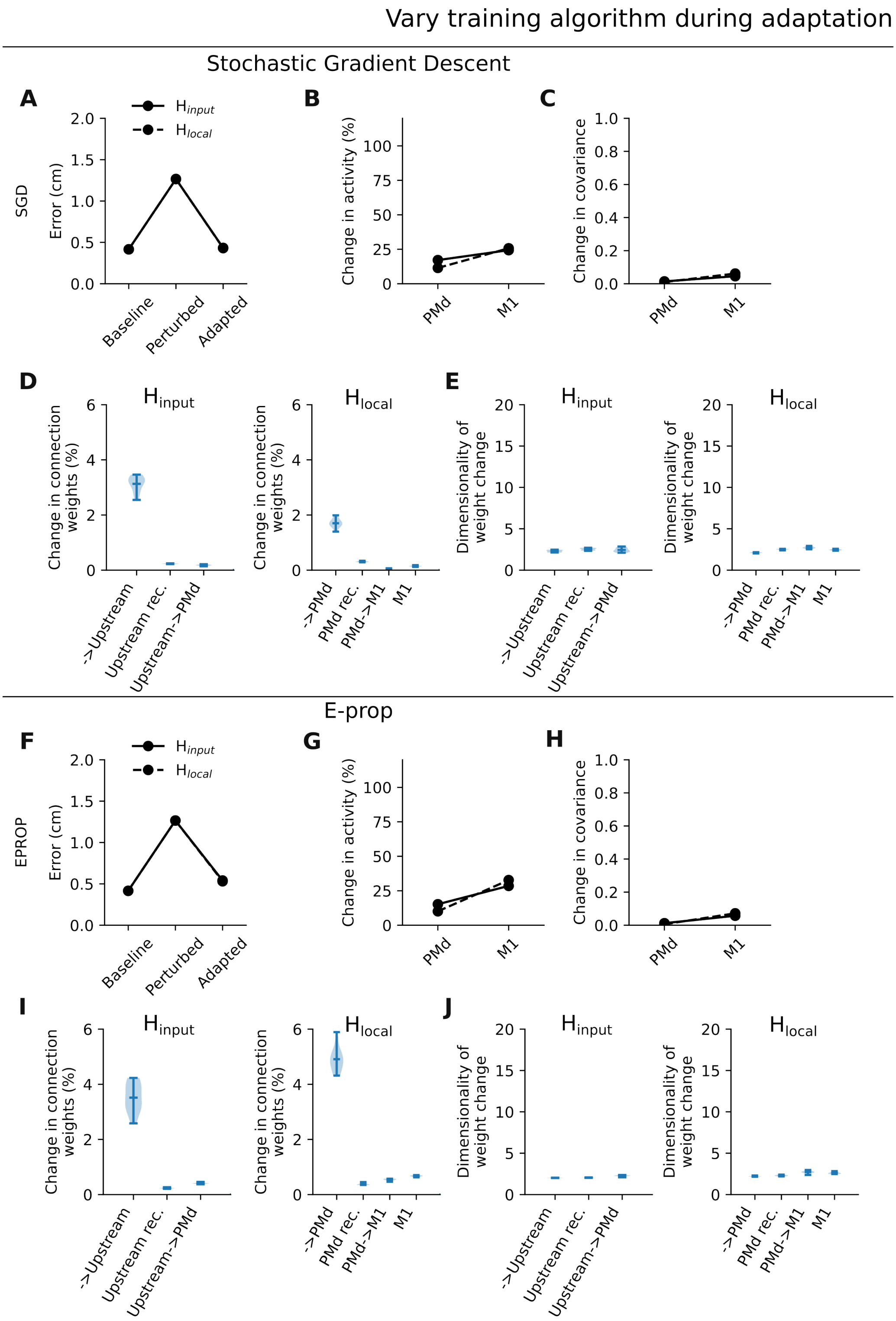

Supplementary Figure 12: Results are robust to variations in training algorithm. We repeated all the adaptation simulations using either Stochastic Gradient Descent **(A-E)** or a recent biologically plausible training algorithm (E-prop) [Bellec et al., 2020] to retrain the network during adaptation **(F-J)**. **A.** Root mean squared error between target and produced hand trajectories after initial training (Baseline), exposure to the VR perturbation (Perturbed), and after adaptation (Adapted) under H_{input} (solid line) and H_{local} (dashed line). Markers and error bars, mean and s.d. (n=10 network initialisations). **B.** Change in trial-averaged activity. **C.** Change in covariance. Data in B,C are presented as in A. **D.** Change in connection weights following adaptation under H_{input} (left) and H_{local} (right). Shaded area and horizontal bars, data distribution with mean and extrema (n=10 network initialisations). **E.** Estimated dimensionality of connection weight changes. Data presented as in D. Data in F,G,H,I,J are presented as in A,B,C,D,E, respectively.

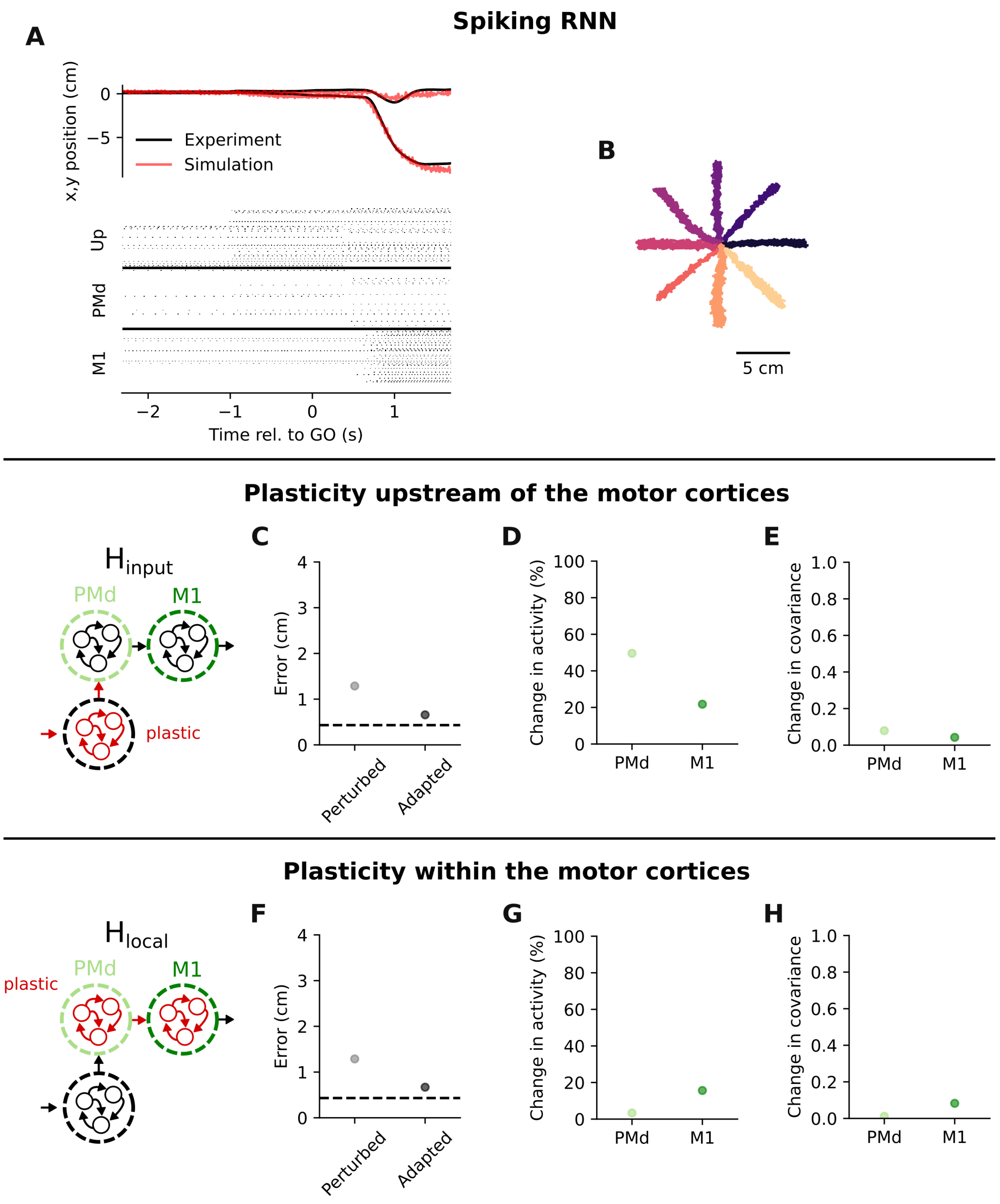

Supplementary Figure 13: A modular spiking neural network replicates the main results obtained using a rate-based network. A. Modular spiking RNN trained with Backpropagation Through Time using Surrogate Gradients [Zenke and Ganguli, 2018, Neftci et al., 2019]. Each module (Upstream, PMd, M1) consisted of 100 neurons, respectively. Top: The produced output matched the experimental data well. Bottom: example neuronal firing for the same example example trial. **B.** Simulated hand trajectories after initial training. **C.** Root mean squared error between target and produced hand trajectories after initial training (dashed line), after the VR perturbation is introduced (grey) and after adaptation to the perturbation under $\mathrm{H}_{\mathrm{input}}$ (black). **D.** Changes in trial-averaged activity following adaptation under $\mathrm{H}_{\mathrm{input}}$ for PMd and M1, respectively. **E.** Change in covariance following adaptation under $\mathrm{H}_{\mathrm{input}}$; data presented as in D. **F.** Root mean squared error between target and produced hand trajectories under $\mathrm{H}_{\mathrm{local}}$. **G.** Changes in trial-averaged activity following adaptation under $\mathrm{H}_{\mathrm{local}}$ for PMd and M1, respectively. **H.** Change in covariance following adaptation under $\mathrm{H}_{\mathrm{local}}$. Data in F,G,H are presented as in C,D,E.

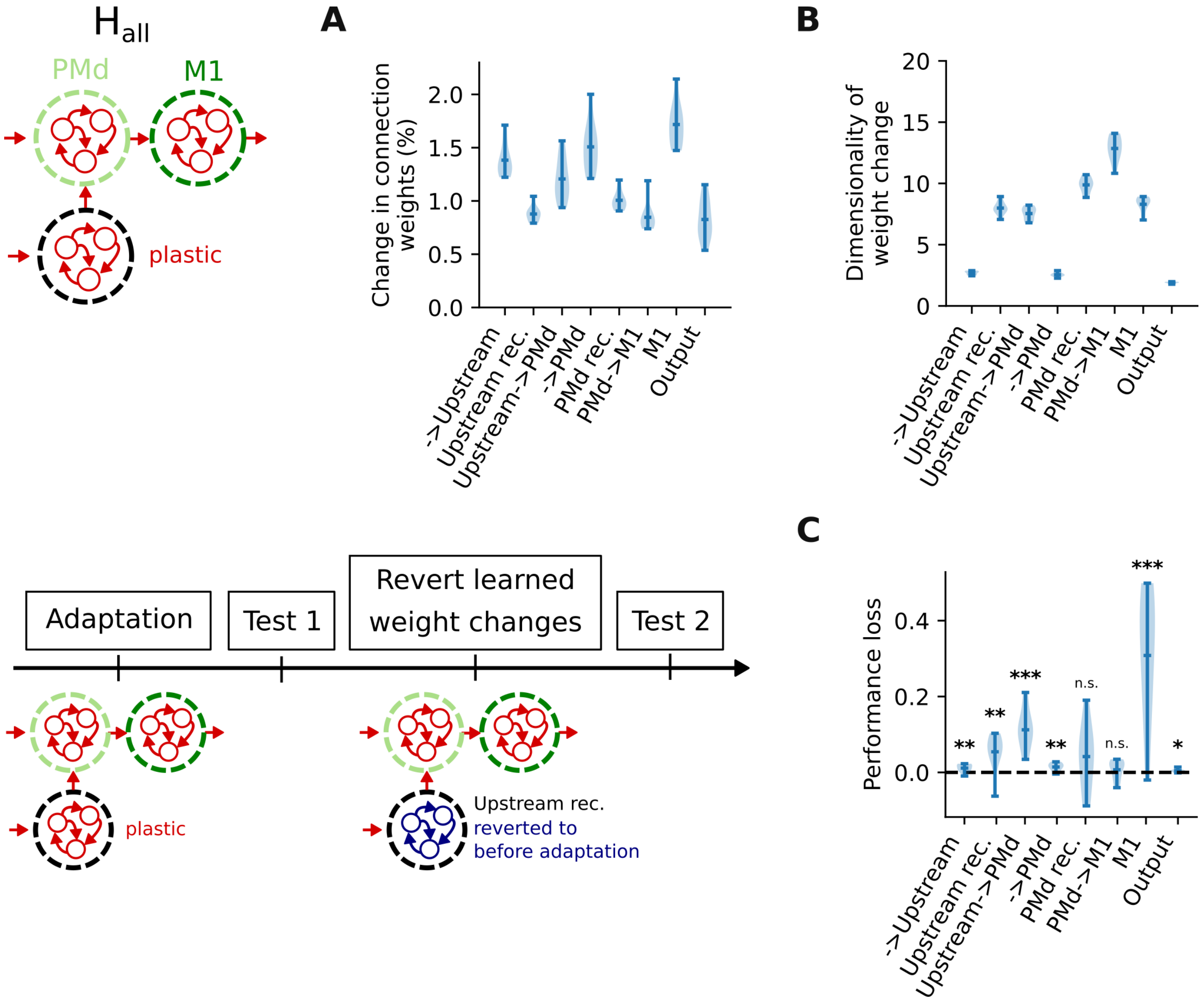

Supplementary Figure 14: Analysis of weight changes for H_{all}. A. Change in connection weights following adaptation under H_{all} (all parameters are allowed to be plastic). Shaded area and horizontal bars, data distribution with mean and extrema (n=10 network initialisations). Same data presentation in all panels. **B.** Estimated dimensionality of connection weight changes for each network module and cross-module connections. **C.** Contribution of the learned within-module or cross-module weight changes to adaptation. Following VR adaptation, we manually set the connectivity values of one module or set of cross-module connections to its corresponding connectivity values before adaptation, and tested how much this impaired motor output. Performance loss is given by the difference in mean squared error between before and after we reverted the learned weight changes (Test 1 versus Test 2 in the schematic). Two-sided t-test was used to test the null hypothesis of zero population mean (scipy package ttest_1samp function) (t=3.32 p=0.0089; t=3.61 p=0.0056; t=6.59 p=0.0001; t=4.12 p=0.0026; t=1.53 p=0.1599; t=0.84 p=0.4236; t=5.77 p=0.0003; t=3.18 p=0.0111; ordered as presented in C).

Supplementary Methods

E-prop learning algorithm (Supplementary Figure 12)

Instead of using the Adam optimizer during the adaptation phase, we also tested a more biologically plausible, online learning rule [Murray, 2019, Bellec et al., 2020]. Each neuron received a weighted error signal about the current difference between produced and target hand position. The weighted error was given by projecting the position error back through the network hierarchy, using the transpose of the feedforward matrices.

$$\begin{aligned} \epsilon &= \mathbf{x}^{out} - \mathbf{x}^{target} & (1) \\ \epsilon^{M1} &= (\mathbf{W}^{out})^T \; (\epsilon - \mathbf{b}^{out}) & (2) \\ \epsilon^{PMd} &= (\mathbf{W}^{PMd-M1})^T \; \epsilon^{M1} & (3) \\ \epsilon^{UP} &= (\mathbf{W}^{UP-PMd})^T \; \epsilon^{PMd} & (4) \end{aligned}$$

The weight update for a single connection from neuron i to neuron j was then given by multiplying the error signal of the postsynaptic neuron j with a term dependent on the activity of the postsynaptic and the presynaptic neuron.

$$dw_t^{ji} = -\eta \; \epsilon_t^j \left(1 - \tanh(x_t^j)^2\right) \sum_{t'<t} \tanh(x_{t'}^i) \tag{5}$$

where $\eta = 0.000001$ and a batch size of one was used. The weight update described above was applied every 0.2s, starting from 0.5s in the trial. Note that the sum above is bounded as we reset the time for every trial. An example weight update for $\mathbf{W}^{UP-PMd}$ would then be

$$dw_t^{PMd,j;\; UP,i} = -\eta \; \epsilon_t^{PMd,j} \left(1 - \tanh(x_t^{PMd,j})^2\right) \sum_{t'<t} \tanh(x_{t'}^{UP,i}) \tag{6}$$

Defining the learning location was then similar to what has been done in the main manuscript.

- $\mathrm{H}_{\text{input}}$: $\mathbf{W}^{in,UP}$, $\mathbf{W}^{UP}$, $\mathbf{W}^{UP-PMd}$ are plastic
- $\mathrm{H}_{\text{local}}$: $\mathbf{W}^{in,PMd}$, $\mathbf{W}^{PMd}$, $\mathbf{W}^{PMd-M1}$, $\mathbf{W}^{M1}$ are plastic

Spiking neural network (Supplementary Figure 13)

To transform our model from a rate-based to a spiking neural network we closely followed a recently published tutorial [Zenke, 2019b,Zenke, 2019a], based on this paper [Neftci et al., 2019]. We mapped the RNN dynamics to a network of leaky-integrate-and-fire neurons, preserving the model architecture described above. We used a membrane time constant of 50ms and a synaptic time constant of 25ms. The readout neurons were modelled as simple integrators with a longer timescale, potentially mimicking slower processes related to muscle activation. For the readout neurons a membrane time constant of 500ms and a synaptic time constant of 200ms. To train the spiking network using the Adam optimizer we used the method of surrogate gradients [Neftci et al., 2019], where the spiking nonlinearity is replaced by a sigmoid for the backward pass of gradient descent. The steepness of that sigmoid was set to 100 (following the tutorial). The stimulus given to the network was defined as before, as a non-spiking, tonic input. The parameters for these simulations were: number of neurons per module = 100, batch size=20, $dt = 0.01$, learning rate=0.001, regularization $\alpha = 0.001$ (here, we used regularization only on the weights, not the rates), gradient clipping at norm 0.2.

Supplementary References

[Bellec et al., 2020] Bellec, G., Scherr, F., Subramoney, A., Hajek, E., Salaj, D., Legenstein, R., and Maass, W. (2020). A solution to the learning dilemma for recurrent networks of spiking neurons. *Nature communications*, 11(1):1–15.

[Murray, 2019] Murray, J. M. (2019). Local online learning in recurrent networks with random feedback. *ELife*, 8:e43299.

[Neftci et al., 2019] Neftci, E. O., Mostafa, H., and Zenke, F. (2019). Surrogate gradient learning in spiking neural networks: Bringing the power of gradient-based optimization to spiking neural networks. *IEEE Signal Processing Magazine*, 36(6):51–63.

[Zenke, 2019a] Zenke, F. (2019a). Spytorch. *Zenodo*.

[Zenke, 2019b] Zenke, F. (2019b). Tutorial 1: Training a spiking neural network with surrogate gradients. `https://github.com/fzenke/spytorch/blob/v0.3/notebooks/SpyTorchTutorial1.ipynb/`.

[Zenke and Ganguli, 2018] Zenke, F. and Ganguli, S. (2018). Superspike: Supervised learning in multilayer spiking neural networks. *Neural computation*, 30(6):1514–1541.
