# Supplementary material for: Small, correlated changes in synaptic connectivity may facilitate rapid motor learning
Source: Nat Commun. 2022 Sep 2;13:5163. doi: 10.1038/s41467-022-32646-w (PMC9440011; doi:10.1038/s41467-022-32646-w)
Supplement: Supplementary file 2 — Reporting Summary [file 41467_2022_32646_MOESM2_ESM.pdf]

nature portfolio

Corresponding author(s): Professor Claudia Clopath

Last updated by author(s): Jun 30, 2022

Reporting Summary

Nature Portfolio wishes to improve the reproducibility of the work that we publish. This form provides structure for consistency and transparency in reporting. For further information on Nature Portfolio policies, see our Editorial Policies and the Editorial Policy Checklist.

Statistics

For all statistical analyses, confirm that the following items are present in the figure legend, table legend, main text, or Methods section.

n/a	Confirmed	
☐	☒	The exact sample size (n) for each experimental group/condition, given as a discrete number and unit of measurement
☐	☒	A statement on whether measurements were taken from distinct samples or whether the same sample was measured repeatedly
☐	☒	The statistical test(s) used AND whether they are one- or two-sided<br>*Only common tests should be described solely by name; describe more complex techniques in the Methods section.*
☒	☐	A description of all covariates tested
☒	☐	A description of any assumptions or corrections, such as tests of normality and adjustment for multiple comparisons
☐	☒	A full description of the statistical parameters including central tendency (e.g. means) or other basic estimates (e.g. regression coefficient) AND variation (e.g. standard deviation) or associated estimates of uncertainty (e.g. confidence intervals)
☐	☒	For null hypothesis testing, the test statistic (e.g. F, t, r) with confidence intervals, effect sizes, degrees of freedom and P value noted<br>*Give P values as exact values whenever suitable.*
☒	☐	For Bayesian analysis, information on the choice of priors and Markov chain Monte Carlo settings
☒	☐	For hierarchical and complex designs, identification of the appropriate level for tests and full reporting of outcomes
☒	☐	Estimates of effect sizes (e.g. Cohen's d, Pearson's r), indicating how they were calculated

Our web collection on statistics for biologists contains articles on many of the points above.

Software and code

Policy information about availability of computer code

Data collection	Custom simulation code was written in python-3.7.4, using the pytorch-1.8.1 package to train the network models. The code will be made publicly available upon publication under https://github.com/babaf/motor-adaptation-local-vs-input.
Data analysis	All data analysis was performed using python-3.7.4; except to fit the linear mixed model for the experimental data presented in Figure 2, where R and the lmer function from the lmerTest package was used. The R version was 4.0.3. .

For manuscripts utilizing custom algorithms or software that are central to the research but not yet described in published literature, software must be made available to editors and reviewers. We strongly encourage code deposition in a community repository (e.g. GitHub). See the Nature Portfolio guidelines for submitting code & software for further information.

Data

Policy information about availability of data

All manuscripts must include a data availability statement. This statement should provide the following information, where applicable:

- Accession codes, unique identifiers, or web links for publicly available datasets
- A description of any restrictions on data availability
- For clinical datasets or third party data, please ensure that the statement adheres to our policy

The data that support the findings in this study are available from the corresponding authors upon reasonable request.

Field-specific reporting

Please select the one below that is the best fit for your research. If you are not sure, read the appropriate sections before making your selection.

☒ Life sciences ☐ Behavioural & social sciences ☐ Ecological, evolutionary & environmental sciences

For a reference copy of the document with all sections, see nature.com/documents/nr-reporting-summary-flat.pdf

Life sciences study design

All studies must disclose on these points even when the disclosure is negative.

Sample size	We analyzed data from four implants and two monkeys monkeys to show reproducibility. Given our recording methods, we could not control the number of neurons studied for this analysis, but we recorded sufficient neurons to extract information from the population. Application of principal component analysis to the binned firing rates identified a few (e.g., 6-10) dominant patterns of neural covariance that explained a substantial amount of total neural variance (typically >50 %). Prior studies have demonstrated accurate reconstruction of population dynamics using neuron counts similar to those we obtained. We simulated ten differently initialized networks to ensure reproducibility across network initialisations.
Data exclusions	For the electrophysiology data, since we used extracellular recordings with chronically implanted electrode arrays, we could not control the specific cells we recorded. Thus, we excluded some cells with low firing rates (< 1 Hz mean firing rate across all bins) or very poor recording quality. The latter was assessed using a combination of: 1) visual identification of threshold crossings waveforms that appeared to be artifacts rather than neural in origin, and 2) an analysis of cross-channel 'shunting' based on calculating cross-correlograms of the binned neural firing rates computed in small (1 ms) bins. No data was excluded from the simulation data analysis.
Replication	We ran several control simulations (cf. supplementary figures) which all qualitatively supported the main findings in the paper. For the electrophysiology data, we reproduced our main findings in 11 sessions from two different monkeys.
Randomization	This allocation was not relevant to our study, since we studied all experimental subjects under similar behavioral conditions. There are no subjects or groups among the models.
Blinding	Blinding was not relevant to this study, since, e.g., we did not seek to compare the outcome of different interventions. There are no subjects or groups among the models.

Reporting for specific materials, systems and methods

We require information from authors about some types of materials, experimental systems and methods used in many studies. Here, indicate whether each material, system or method listed is relevant to your study. If you are not sure if a list item applies to your research, read the appropriate section before selecting a response.

Materials & experimental systems

n/a	Involved in the study	
☒	☐	Antibodies
☒	☐	Eukaryotic cell lines
☒	☐	Palaeontology and archaeology
☐	☒	Animals and other organisms
☒	☐	Human research participants
☒	☐	Clinical data
☒	☐	Dual use research of concern

Methods

n/a	Involved in the study	
☒	☐	ChIP-seq
☒	☐	Flow cytometry
☒	☐	MRI-based neuroimaging

Animals and other organisms

Policy information about studies involving animals; ARRIVE guidelines recommended for reporting animal research

Laboratory animals	This study used two monkeys (all male, macaca mulatta). Monkey C was 5-8 years old, Monkey M was 6-7 years old.
Wild animals	This study did not involve wild animals.
Field-collected samples	This study did not involve samples collected in the field.
Ethics oversight	All surgical and experimental procedures were approved by the Institutional Animal Care and Use Committee (IACUC) of Northwestern University.

Note that full information on the approval of the study protocol must also be provided in the manuscript.
